# Chronic disease outcome metadata from German observational studies – public availability and FAIR principles

**DOI:** 10.1038/s41597-023-02726-7

**Published:** 2023-12-05

**Authors:** Carolina Schwedhelm, Katharina Nimptsch, Wolfgang Ahrens, Hans Martin Hasselhorn, Karl-Heinz Jöckel, Verena Katzke, Alexander Kluttig, Birgit Linkohr, Rafael Mikolajczyk, Ute Nöthlings, Ines Perrar, Annette Peters, Carsten O. Schmidt, Börge Schmidt, Matthias B. Schulze, Andreas Stang, Hajo Zeeb, Tobias Pischon

**Affiliations:** 1 Molecular Epidemiology Research Group, Max Delbrück Center for Molecular Medicine in the Helmholtz Association (MDC), Berlin, 13125 Germany; 2 Leibniz Institute for Prevention Research and Epidemiology – BIPS, Bremen, 28359 Germany; 3 Institute of Statistics, Faculty of Mathematics and Computer Science, University of Bremen, Bremen, 28334 Germany; 4 Department of Occupational Health Science, University of Wuppertal, Wuppertal, 42119 Germany; 5 Institute for Medical Informatics, Biometry and Epidemiology, University Hospital of Essen, Essen, 45122 Germany; 6 Division of Cancer Epidemiology, German Cancer Research Center (DKFZ), Heidelberg, 69120 Germany; 7 Institute of Medical Epidemiology, Biometrics, and Informatics, Interdisciplinary Center for Health Sciences, Medical Faculty of the Martin-Luther-University Halle-Wittenberg, Halle (Saale), 06112 Germany; 8 Institute of Epidemiology, Helmholtz Zentrum München, German Research Center for Environmental Health, Neuherberg, 85764 Germany; 9 DZPG (German Center for Mental Health), partner site Halle-Jena-Magdeburg, 07743 Jena, Germany; 10 Institute of Nutrition and Food Sciences, Nutritional Epidemiology, University of Bonn, Bonn, 53115 Germany; 11 Institute for Medical Information Processing, Biometry and Epidemiology, Department of Epidemiology, Medical Faculty of the Ludwig-Maximilians-Universität München, Munich, 81377 Germany; 12 Institute for Community Medicine, University Medicine Greifswald, Greifswald, 17489 Germany; 13 Department of Molecular Epidemiology, German Institute of Human Nutrition Potsdam Rehbruecke, Nuthetal, 14558 Germany; 14 Institute of Nutritional Science, University of Potsdam, Nuthetal, 14558 Germany; 15 Department of Epidemiology, School of Public Health, Boston University, Boston, MA 02118 USA; 16 Faculty 11 - Human and Health Sciences, University of Bremen, Bremen, 28359 Germany; 17 Biobank Technology Platform, Max-Delbrueck-Center for Molecular Medicine in the Helmholtz Association (MDC), Berlin, 13125 Germany; 18 Core Facility Biobank, Berlin Institute of Health at Charité – Universitätsmedizin Berlin, Berlin, 13125 Germany; 19 Charité – Universitätsmedizin Berlin, Corporate Member of Freie Universität Berlin and Humboldt-Universität zu Berlin, Berlin, 10117 Germany

**Keywords:** Cardiovascular diseases, Cancer, Metabolic disorders, Research data

## Abstract

Metadata from epidemiological studies, including chronic disease outcome metadata (CDOM), are important to be findable to allow interpretability and reusability. We propose a comprehensive metadata schema and used it to assess public availability and findability of CDOM from German population-based observational studies participating in the consortium National Research Data Infrastructure for Personal Health Data (NFDI4Health). Additionally, principal investigators from the included studies completed a checklist evaluating consistency with FAIR principles (Findability, Accessibility, Interoperability, Reusability) within their studies. Overall, six of sixteen studies had complete publicly available CDOM. The most frequent CDOM source was scientific publications and the most frequently missing metadata were availability of codes of the International Classification of Diseases, Tenth Revision (ICD-10). Principal investigators’ main perceived barriers for consistency with FAIR principles were limited human and financial resources. Our results reveal that CDOM from German population-based studies have incomplete availability and limited findability. There is a need to make CDOM publicly available in searchable platforms or metadata catalogues to improve their FAIRness, which requires human and financial resources.

## Introduction

Numerous epidemiological studies have been examining risk factors of chronic diseases such as cancer, cardiovascular diseases, and diabetes, which represent a high burden of disease globally^1,2^. In Germany, where these three disease groups account for 44% of the total disability-adjusted life years (19.5%, 18.8%, 5.8% for cancer, cardiovascular diseases and diabetes, respectively in 2019)^3^, several population-based observational studies are dedicated to the study of risk factors of chronic diseases. The potential of research data derived by these studies to improve our understanding of health and disease can be substantially enhanced by following the FAIR principles (Findability, Accessibility, Interoperability, Reusability), optimizing interpretability and reproducibility of results, as well as reuse of data^4^. While it is increasingly accepted that all research data should follow the FAIR principles, implementation is not ubiquitous and interoperability across data sources is still limited^5,6^. In Germany, the consortium National Research Data Infrastructure for Personal Health Data (NFDI4Health, https://www.nfdi4health.de/en/) - with the participation of 26 observational studies – seeks to increase the value of research in epidemiology, public health, and clinical trial-based medicine, by making high quality personal health research data from Germany internationally accessible according to the FAIR principles^7^.

Research data in population-based observational studies usually refers to the sum of data that characterize each participant in that study, or parts thereof (i.e., personal data, unless anonymized). However, an important step in achieving FAIR data is the availability of rich metadata describing these research data^8^. The assessment of chronic diseases in observational studies is challenging, and each disease can be assessed in many different ways. Thus, the methods used to assess diseases differ between studies, depending on study aims, design, study population, and resources available^9^. In the case of chronic diseases, metadata include, among others, information on whether the outcome is prevalent or incident, on disease subtypes assessed and classification system(s) used, how data were collected (i.e., questionnaires, interviews, study examinations, administrative databases, or through a combination of sources), and whether and how self-reported diseases were verified (i.e., confirmed on a case basis) or validated (i.e., plausibility of prevalence or incidence observed in a study population evaluated based on a reference population)^10^.

Differences in assessment methods used have implications on how the data can be reused and how they should be interpreted. Knowledge of how data are collected is not only important for the scientific community to gain awareness of contextual constraints impacting interpretation, but also to enable reuse of data, for example in meta-analyses or pooled analyses. However, details on chronic disease assessment methods – hereafter referred to as chronic disease outcome metadata (CDOM) – are often difficult to find. Therefore, there is a need for specific reporting guidelines using a common metadata schema capturing the vast characteristics of chronic disease assessment and ascertainment methods used in epidemiological studies.

This study proposes a schema for CDOM in epidemiological studies and applies it to population-based observational studies in Germany, describing the current status of CDOM public availability and findability. Additionally, it assesses perceived consistency of CDOM with FAIR principles within identified studies.

## Results

### Summary of included studies

Sixteen observational studies participating in NFDI4Health collected chronic disease data (i.e., data on cardiovascular diseases, cancer, and/or type 2 diabetes mellitus). Of these, most studies had a cohort design (n = 13, with sample size ranging from 1,779 to ~205,000 participants), one was a cross-sectional study (7,124 participants), one had a mixed design with both cross-sectional and cohort characteristics (sample size of 8,152 participants), and one study comprised of multiple cross-sectional surveys (four samples ranging from 19,294 to 24,016 participants). An overview of the included studies is shown in Table 1. CDOM for these studies were searched according to the search strategy and criteria described in the methods section and in Supplementary Table 1.

**Table 1:** Overview of included German population-based observational studies (n = 16).

Study name	Study design	Sample size	Recruitment years	Age at recruitment
CARLA (*Cardiovascular Disease, Living and Ageing in Halle*)	Cohort	1,779	2002–2006	45–83 y
DEGS1^a^ (*German Health Interview and Examination Survey for Adults*)	Mixed (cross-sectional and cohort)	8,152	2008–2011	18–79 y
DONALD (*Dortmund Nutritional and Anthropometric Longitudinally Designed Study*)	(Open) cohort	~2,300^b^	since 1985 (ongoing)	3 mo
EPIC-Heidelberg (*European Prospective Investigation into Cancer and Nutrition – Heidelberg cohort*)	Cohort	25,540	1994–1998	35–65 y
EPIC-Potsdam (*European Prospective Investigation into Cancer and Nutrition – Potsdam cohort*)	Cohort	27,548	1994–1998	35–65 y
GEDA – multiple studies (2009–2021) (*German Health Update*)	Cross-sectional	21,262	2009	18–79 + y
		22,050	2010	
		19,294	2012	
		24,016	2014–2015	
GHS (*Gutenberg Health Study*)	Cohort	15,010	2007–2012	35–74 y
GNHIES98 (BGS98) (*German National Health Interview and Examination Survey 1998*)	Cross-sectional	7,124	1997–1999	18–79 y
HCHS (*Hamburg City Health Study*)	Cohort	~45,000^b^	since 2015 (ongoing)	45–74 y
HNRS (*Heinz Nixdorf Recall Study*)	Cohort	4,814	2000–2003	45–75 y
IDEFICS/I.Family (*Identification and prevention of dietary- and lifestyle-induced health effects in children and infants/Determinants of eating behaviour in European children, adolescents and their parents*)	Cohort	16,228 (IDEFICS)	2007–2008	2–10 y
		9,617 (I.Family)		
KORA (*Cooperative Health Research in the Region of Augsburg*)	Cohort	S1: 4,022	S1: 1984–1985	S1: 25–64 y;
		S2: 4,940	S2: 1989–1990	
		S3: 4,856	S3: 1994–1995	
		S4: 4,261	S4: 1999–2001	S2-4: 25–74 y
lidA (*German Cohort Study on Work, Age, Health and Work Participation*)	Cohort	6,585	2011	46 and 52 y
LIFE-Adult (*Leipzig Research Centre for Civilization Diseases – Adult study*)	Cohort	10,000	2011–2014	18–80 y
NAKO (*German National Cohort; GNC/NAKO*)	Cohort	>205,000^c^	2014–2019	20–69 y
SHIP/SHIP Trend (*Study of Health in Pomerania*)	Cohort	4,308/ 4,420	1997–2001/ 2008–2012	20–79 y

mo, months; y, years.

^a^GNHIES98 participants were invited again for DEGS1.

^b^Ongoing recruitment.

^c^Exact number of participants not yet published.

### Publication of chronic disease outcome metadata: evaluation based on proposed schema

A metadata schema with all relevant CDOM was developed within NFDI4Health (see Table 2). CDOM were evaluated in each study per source and outcome and considered to be complete when information about all CDOM fields was available (metadata sources and metadata completeness evaluation scheme described in Tables 3, 4, respectively). For this, an in-depth search within the identified sources of metadata was performed and the identified metadata was recorded in detail by source and metadata field in Supplementary Table 2. This information was then used to summarize our findings in Tables 5, 6, described in the following results subsections. More details are provided in the methods section. Out of the sixteen included studies, publicly available CDOM were *complete for all outcomes* for 6 studies (CARLA, GEDA, NAKO, KORA, lidA, SHIP/SHIP Trend), *complete for some outcomes* for 4 studies (EPIC-Heidelberg, EPIC-Potsdam, GHS, IDEFICS/I.Family), and *partial* for the remaining 6 studies. Table 5 shows the overall status of publicly available CDOM in each study.

**Table 2:** Chronic disease outcome metadata schema.

1. **General information**
a. Prevalent or incident disease outcome I.e., list of chronic disease outcomes examined in the study, differentiating also between prevalent and incident disease outcomes.
b. Classification system used for the chronic diseases, generally codes of the International Classification of Diseases, Tenth Revision (ICD-10)
c. Primary or secondary outcome within the study
2. **Assessment method: collection method**
a. Self-report i. Questionnaire/interview mode and device I.e., self-completed: paper-based or computer-based; face-to-face: computer-assisted personal interview (CAPI) or face-to-face paper-based interview; telephone: computer-assisted telephone interview (CATI), paper-based telephone interview. ii. Disease domain(s) E.g., questions about disease, diagnosis, symptoms, and/or treatment/medication. iii. Reference period E.g., questions referring to the domain: current, last month, last 6 months, last 12 months, ever. iv. Verification of individual cases and/or additional external validation E.g., verification methods: hospital/treatment documentation provided by participant, treating physician, hospital/medical records, health insurance, disease registry, death certificate. E.g., external validation methods: validation study comparing prevalence/incidence plausibility against a random subsample or a standard, such as medical records of the source population).
b. Study examinations i. Which tests/examinations, including procedures and cut-offs/thresholds E.g., blood pressure measurements for hypertension as outcome: three consecutive blood pressure measurements 3 minutes apart. Hypertension if mean systolic blood pressure ≥ 140 mmHg and/or mean diastolic blood pressure ≥ 90 mmHg, and/or use of antihypertensive medication according to ATC code, given the participant had known hypertension.
c. Administrative databases i. Source(s) E.g., health insurance, disease registry, death certificate.

**Table 3:** Sources of published outcome metadata^a^.

1. **Scientific publications** Descriptive publications (e.g., cohort profile/data resource profile, protocol describing study objectives and design) and analytic publications (i.e., focusing on specific research question(s)) in scientific journals.
2. **Study websites** Descriptions of the study and procedures.
3. **Study/trial registries** Descriptions of the study (e.g., study/trial registries like clinicaltrials.gov, metadata repositories).
4. **Data documents** Study reports, data dictionaries, lists of variables, questionnaires, etc. Data documents are often available through (meta-)data access infrastructure (i.e., web portals).

^a^Adapted from previously defined sources contributing to (meta-)data discoverability (McMahon 2017, https://discovery.ucl.ac.uk/id/eprint/10025205)^25^

**Table 4:** Evaluation scheme of studies’ completeness of publicly accessible chronic disease outcome metadata.

Classification	Description	
	Evaluation applied to the study	Evaluation applied by metadata field
Complete metadata for all outcomes	All metadata fields from Table 2 can be obtained for all examined chronic disease outcomes based on publicly accessible metadata.	A complete description of this metadata field was found for all examined chronic disease outcomes.
		Score: 3 points.
Complete metadata for some outcomes	All metadata fields from Table 2 can be obtained for some but not all examined chronic disease outcomes based on publicly accessible metadata.	A complete description of this metadata field was found for some but not all examined chronic disease outcomes.
		Score: 2 points.
Partial metadata	Some metadata fields from Table 2 can be obtained for all or some of the examined chronic disease outcomes based on publicly accessible metadata.	A partial description of this metadata field was found for all or some of the examined chronic disease outcomes (details are missing).
		Score: 1 point.
Metadata missing	None of the metadata fields from Table 2 can be obtained based on publicly accessible metadata.	Nothing describing this metadata field was found for any of the examined chronic disease outcomes.
		Score: 0 points.

**Table 5:** Published chronic disease outcome metadata in the included studies (n = 16)^a^.

Study	Publicly available chronic disease outcome metadata by source				(Meta-)data access infrastructure	Overall status
	Scientific publications	Study website	Study/trial registries	Data documents		
CARLA	All metadata described for some outcomes^26–31^	All metadata described (incl. links to scientific publications, data documents)^32^	Partial metadata described^33^	Data dictionaries; partial metadata described^34^	Availability: yes; Accessibility: metadata (data dictionary) accessible without registration^32^	Complete metadata for all outcomes
DEGS1	Partial metadata described^30,35,36^	Partial metadata described; links to scientific publications and data documents^37^	Not found	Variable list for all prevalent outcomes (baseline only); partial metadata described^38^	Availability: yes; Accessibility: metadata (DEGS1 data dictionary) accessible without registration^38^	Partial metadata
DONALD	Partial metadata described^39–48^	Partial metadata described; links to scientific publications^49^	Partial metadata described; links to website and scientific publications^50–53^	Not found	Availability: no	Partial metadata
EPIC-Heidelberg	All metadata described for some outcomes^30,54–65^	Partial metadata described; links to scientific publications^66,67^	Not found	Not found	Availability: no; application process to obtain (meta-)data is described^68^	Complete metadata for some outcomes
EPIC- Potsdam	All metadata described for some outcomes^30,55,56,69–77^	Partial metadata described; links to scientific publications^67,78^	Partial metadata described; links to scientific publications^50–53^	Not found	Availability: no; application process to obtain data is described^68^	Complete metadata for some outcomes
GEDA 2009, 2010, 2012, 2014/2015, 2019/2020, 2021	All metadata described; questionnaires available as annex^79–82^	Partial metadata described; links to scientific publications and data documents^83,84^	Not found	Variable list and questionnaire up to 2019/2020 with all metadata described^38,79,82^	Availability: yes; Accessibility: metadata (data dictionary) accessible without registration^38^	Complete metadata for all outcomes
GHS	All metadata described for some outcomes^30,85–100^	Partial metadata described^101^	Not found	Not found	Availability: no	Complete metadata for some outcomes
GNHIES98 (BGS98)	Partial metadata described^102^	Partial metadata described; links to scientific publications and data documents^103^	Not found	Variable list with partial metadata described^38^	Availability: yes; Accessibility: metadata (data dictionary) accessible without registration^38^	Partial metadata
HCHS	Partial metadata described^104–107^	No metadata described; links to scientific publications^105,108^	Partial metadata described; links to website, scientific publications^109^	Not found	Availability: yes; Accessibility: no (credentials needed; no registration option)^110^	Partial metadata
HNRS	Partial metadata described^30,111–122^	Partial metadata described^123^	Partial metadata described; links to scientific publications^124^	Not found	Availability: no	Partial metadata
IDEFICS/ I.Family	All metadata described for some outcomes^116,125–128^	Partial metadata described; links to scientific publications^129–131^	Partial metadata described; links to scientific publications^51,132,133^	SOPs with partial metadata described^125,134^	Availability: yes; Accessibility: metadata (SOPs, questionnaires) accessible after guest registration (temporary access)^134^	Complete metadata for some outcomes
KORA	Partial metadata described refs. ^30,135–144^	Partial metadata described; links to scientific publications ref. ^145^	Partial metadata described ref. ^146^	Variable lists with partial metadata described ref. ^147^	Availability: yes; Accessibility: metadata (variable lists) accessible after registration ref. ^147^	Complete metadata for all outcomes
lidA	Partial metadata described^148^	Partial metadata described; links to scientific publications^149^	Not found	Data reports with partial metadata^150^	Availability: yes; Accessibility: metadata (reports with variable descriptions) accessible without registration^150^	Complete metadata for all outcomes
LIFE-Adult	Partial metadata described^151–153^	Partial metadata described; links to scientific publications^154^	Partial metadata described; links to study website^155^	Data dictionaries with partial metadata^156^	Availability: yes; Accessibility: metadata (data dictionaries) accessible after registration^156^	Partial metadata
NAKO	All metadata described for all outcomes^157–161^	Partial metadata described; links to scientific publications^162^	Partial metadata described; links to study website linking to scientific publications^163^	Data dictionary with some metadata described^164^	Availability: yes; Accessibility: metadata (data dictionary) accessible without registration; registration is also possible^164^	Complete metadata for all outcomes
SHIP/SHIP Trend	Partial metadata described^30,165–182^	Partial metadata described; links to scientific publications and data documents^183^	Partial metadata described; links to scientific publications and study website^33,53,146,163,184–186^	Interview and study examination forms, data dictionaries with partial metadata described^183,187^	Availability: yes; Accessibility: metadata (data dictionaries) accessible without registration^187^	Complete metadata for all outcomes

^a^Metadata considered complete if all aspects of the chronic disease outcome metadata schema (Table 2) are covered for all examined cardiovascular diseases, type 2 diabetes, and cancers; metadata complete for “all outcomes” refers to the evaluation of these diseases only.

**Table 6:** Completeness of public available chronic disease outcome metadata (by source and overall)^a^.

Study	Source of metadata	General information			Assessment method						Overall status per study
		Prevalent/ incident outcome	ICD-10 available	Primary/ secondary outcome	Self-report				Study examinations	Administrative databases^b^	
					Mode & Device	Domain	Reference period	Verification/ ext.validation			
CARLA	Scientific publications	2	2	3	3	2	2	3	3	3	**Complete metadata for all outcomes**
	Study website	1 (3)	0 (3)	3 (3)	0 (3)	0 (3)	0 (3)	0 (3)	0 (3)	0 (3)	
	Study/trial registries	0 (2)	0 (0)	0 (0)	0 (0)	0 (1)	0 (1)	0 (0)	1 (2)	0 (0)	
	Data documents	3	3	0	3	3	3	3	3	0	
	**Overall**	**3**	**3**	**3**	**3**	**3**	**3**	**3**	**3**	**3**	
DEGS1	Scientific publications	2	0	3	3	3	3	0	3	na.	**Partial metadata**
	Study website	1 (2)	0 (0)	3 (3)	1 (3)	2 (3)	1 (3)	0 (0)	1 (3)	na.	
	Study/trial registries	na.	na.	na.	na.	na.	na.	na.	na.	na.	
	Data documents	2	0	0	0	3	3	0	3	na.	
	**Overall**	**2**	**0**	**3**	**3**	**3**	**3**	**0**	**3**	na.	
DONALD	Scientific publications	3	3	3	2	3	3	0	3	na.	**Partial metadata**
	Study website	3 (3)	0 (3)	3 (3)	0 (2)	2 (3)	3 (3)	0 (0)	2 (2)	na.	
	Study/trial registries	3 (3)	0 (3)	3 (3)	3 (3)	0 (2)	0 (3)	0 (0)	2 (2)	na.	
	Data documents	na.	na.	na.	na.	na.	na.	na.	na.	na.	
	**Overall**	**3**	**3**	**3**	**3**	**3**	**3**	**0**	**3**	na.	
EPIC-Heidelberg	Scientific publications	3	2	3	3	3	2	3	3	3	**Complete metadata for some outcomes**
	Study website	2 (3)	0 (0)	0 (3)	0 (3)	3 (3)	1 (2)	1 (3)	1 (3)	2 (3)	
	Study/trial registries	na.	na.	na.	na.	na.	na.	na.	na.	na.	
	Data documents	na.	na.	na.	na.	na.	na.	na.	na.	na.	
	**Overall**	**3**	**2**	**3**	**3**	**3**	**2**	**3**	**3**	**3**	
EPIC-Potsdam	Scientific publications	3	2	3	3	3	2	3	3	3	**Complete metadata for some outcomes**
	Study website	2 (2)	0 (0)	0 (3)	2 (3)	1 (3)	1 (1)	3 (3)	1 (3)	1 (3)	
	Study/trial registries	2 (2)	2 (2)	3 (3)	1 (1)	3 (3)	0 (1)	1 (1)	1 (3)	1 (1)	
	Data documents	na.	na.	na.	na.	na.	na.	na.	na.	na.	
	**Overall**	**3**	**2**	**3**	**3**	**3**	**2**	**3**	**3**	**3**	
GEDA 2009, 2010, 2012, 2014/2015, 2019/2020, 2021	Scientific publications	3	na.	3	3	3	3	na.	na.	na.	**Complete metadata for all outcomes**
	Study website	1 (3)	na.	3 (3)	3 (3)	0 (3)	0 (3)	na.	na.	na.	
	Study/trial registries	na.	na.	na.	na.	na.	na.	na.	na.	na.	
	Data documents	3	na.	0	0	3	3	na.	na.	na.	
	**Overall**	**3**	na.	**3**	**3**	**3**	**3**	na.	na.	na.	
GHS	Scientific publications	3	2	3	3	3	3	3	3	3	**Complete metadata for some outcomes**
	Study website	0 (0)	0 (0)	3 (3)	3 (3)	1 (1)	1 (1)	0 (0)	2 (2)	0 (0)	
	Study/trial registries	0 (0)	0 (0)	3 (3)	0 (0)	0 (1)	0 (1)	0 (0)	0 (2)	0 (0)	
	Data documents	na.	na.	na.	na.	na.	na.	na.	na.	na.	
	**Overall**	**3**	**2**	**3**	**3**	**3**	**3**	**3**	**3**	**3**	
GNHIES98 (BGS98)	Scientific publications	2	0	3	2	2	2	2	2	na.	**Partial metadata**
	Study website	0 (2)	0 (0)	3 (3)	2 (2)	0 (2)	0 (2)	0 (0)	0 (2)	na.	
	Study/trial registries	na.	na.	na.	na.	na.	na.	na.	na.	na.	
	Data documents	3	0	0	0	3	3	0	1	na.	
	**Overall**	**3**	**0**	**3**	**2**	**3**	**3**	**2**	**2**	na.	
HCHS	Scientific publications	3	0	3	3	1	0	3	2	3	**Partial metadata**
	Study website	0 (3)	0 (0)	0 (3)	0 (3)	0 (1)	0 (0)	0 (3)	0 (2)	0 (3)	
	Study/trial registries	2 (3)	0 (0)	3 (3)	1 (3)	0 (1)	0 (0)	0 (3)	1 (2)	0 (3)	
	Data documents	na.	na.	na.	na.	na.	na.	na.	na.	na.	
	**Overall**	**3**	**0**	**3**	**3**	**1**	**0**	**3**	**2**	**3**	
HNRS	Scientific publications	3	2	3	3	2	1	3	3	3	**Partial metadata**
	Study website	0 (0)	0 (0)	2 (0)	0 (0)	0 (0)	0 (0)	0 (0)	0 (0)	0 (0)	
	Study/trial registries	0 (2)	0 (0)	3 (3)	0 (2)	1 (1)	0 (1)	0 (1)	1 (2)	1 (1)	
	Data documents.	na.	na.	na.	na.	na.	na.	na.	na.	na.	
	**Overall**	**3**	**2**	**3**	**3**	**2**	**1**	**3**	**3**	**3**	
IDEFICS/ I.Family	Scientific publications	3	3	3	3	3	3	2	3	3	**Complete metadata for some outcomes**
	Study website	1 (3)	0 (3)	0 (3)	0 (3)	0 (3)	0 (0)	0 (2)	1 (3)	0 (3)	
	Study/trial registries	2 (3)	0 (3)	3 (3)	0 (3)	0 (3)	0 (3)	0 (2)	1 (3)	1 (3)	
	Data documents	3	0	0	3	3	3	0	3	0	
	**Overall**	**3**	**3**	**3**	**3**	**3**	**3**	**2**	**3**	**3**	
KORA	Scientific publications	3	2	3	3	2	2	2	3	3	**Complete metadata for all outcomes**
	Study website	1 (2)	0 (2)	3 (3)	1 (3)	0 (2)	0 (2)	0 (2)	1 (2)	0 (3)	
	Study/trial registries	3 (3)	3 (3)	2 (3)	1 (3)	3 (3)	2 (2)	3 (3)	1 (2)	1 (3)	
	Data documents	3	1	0	1	3	3	2	2	0	
	**Overall**	**3**	**3**	**3**	**3**	**3**	**3**	**3**	**3**	**3**	
lidA	Scientific publications	0	3	3	3	0	0	3	na.	3	**Complete metadata for all outcomes**
	Study website	0 (0)	0 (3)	0 (3)	1 (3)	0 (0)	0 (0)	0 (3)	na.	3 (3)	
	Study/trial registries	na.	na.	na.	na.	na.	na.	na.	na.	na.	
	Data documents	3	0	0	3	3	3	0	na.	0	
	**Overall**	**3**	**3**	**3**	**3**	**3**	**3**	**3**	na.	**3**	
LIFE-Adult	Scientific publications	2	0	3	3	3	3	3	3	3	**Partial metadata**
	Study website	0 (2)	0 (0)	2 (3)	1 (2)	0 (2)	0 (1)	0 (2)	1 (2)	0 (0)	
	Study/trial registries	0 (2)	0 (0)	3 (3)	0 (2)	0 (2)	0 (1)	0 (2)	0 (2)	0 (0)	
	Data documents	3	0	0	1	3	3	0	3	0	
	**Overall**	**3**	**0**	**3**	**3**	**3**	**3**	**3**	**3**	**3**	
NAKO	Scientific publications	3	3	3	3	3	3	3	3	3	**Complete metadata for all outcomes**
	Study website	0 (3)	0 (3)	3 (3)	1 (3)	0 (3)	0 (3)	3 (3)	1 (3)	0 (3)	
	Study/trial registries	2 (3)	0 (3)	3 (3)	0 (3)	0 (3)	0 (3)	0 (3)	1 (3)	2 (3)	
	Data documents	3	2	0	1	3	3	0	3	0	
	**Overall**	**3**	**3**	**3**	**3**	**3**	**3**	**3**	**3**	**3**	
SHIP/SHIP Trend	Scientific publications	3	3	3	3	3	1	3	3	3	**Complete metadata for all outcomes**
	Study website	3 (3)	0 (0)	3 (3)	0 (2)	0 (2)	0 (2)	0 (3)	0 (3)	0 (0)	
	Study/trial registries	3 (3)	2 (2)	3 (3)	2 (3)	3 (3)	3 (3)	2 (3)	1 (3)	3 (3)	
	Data documents	3	2	0	2	3	3	0	1	**0**	
	**Overall**	**3**	**3**	**3**	**3**	**3**	**3**	**3**	**3**	**3**	
**Median score per metadata field**^c^		**3**	**2**	**3**	**3**	**3**	**3**	**3**	**3**	**3**	

“3”, complete metadata for all outcomes; “2”, complete metadata for some outcomes; “1”, partial metadata for some or all outcomes; “0”, missing metadata; “na.”, not applicable (due to study design or absence of metadata source). Numbers in parentheses represent metadata availability from both direct sources of metadata (embedded in the corresponding source) and indirect sources of metadata (available through links and references).

^a^Metadata considered complete if all aspects of the chronic disease outcome metadata schema (Table 2) are covered for all examined cardiovascular diseases, type 2 diabetes, and cancers; metadata complete for “all outcomes” refers to the evaluation of these diseases only.

^b^Not considered if consulted for case verification only; considered if may be consulted for or complemented disease ascertainment (e.g., cause of death from death certificates to complement disease incidence data).

^c^Median score (range 0–3) per study, to be interpreted as median public availability of chronic disease outcome metadata in the included studies; e.g., 3 = complete for all outcomes, 2 = complete for some outcomes.

#### Public availability by source

**Fig. 1:**
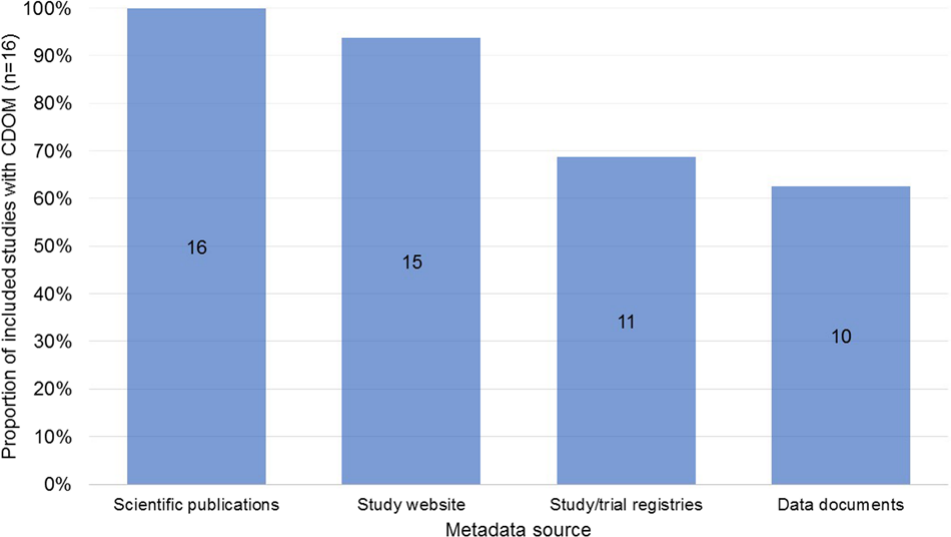
Proportion and number of included studies with publicly available chronic disease outcome metadata (CDOM), by source (total n = 16). Only direct sources of metadata (i.e., links and references not included).

**Fig. 2:**
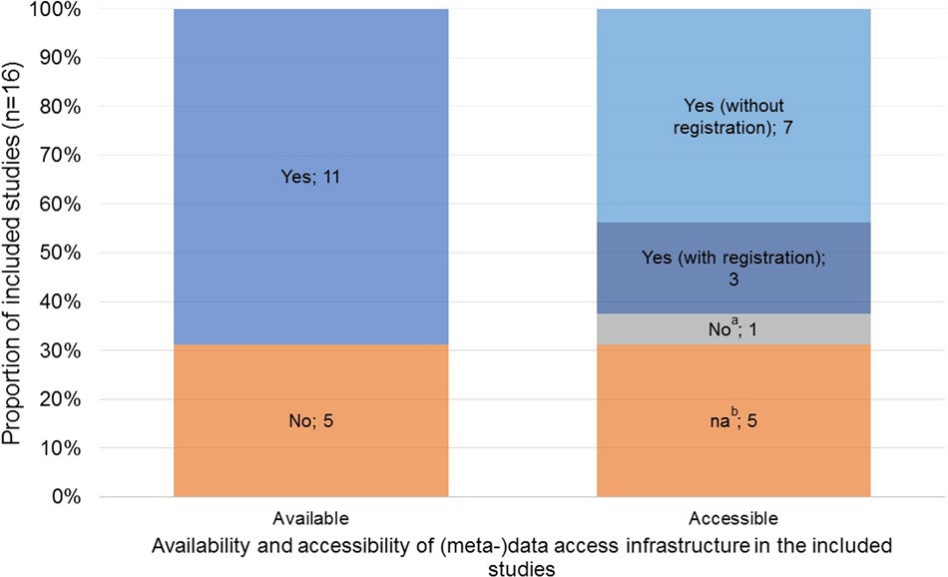
Proportion and number of included studies with available and accessible (meta-)data infrastructure (total n = 16). Study-specific internet-accessible portals (through which data documents are often accessible) were considered as (meta-)data infrastructure. Available if the existence of a (meta-)data infrastructure was identified through the study website and/or data document search; accessible if contents could be viewed without registration or registration. (**a**) Credentials needed, no registration option. (**b**) Corresponding to (meta-)data access infrastructure not available.

Overall, scientific publications were the most frequent source of publicly available CDOM (n = 16), followed by study websites (n = 15; excluding links and references), study/trial registry databases (n = 11; excluding links and references), and data documentation (n = 10) (Fig. 1). Among the six studies with complete publicly available outcome metadata, the main sources of CDOM were scientific publications (GEDA, NAKO, SHIP/SHIP-Trend) and complementary information obtained both through scientific publications and data documentation (CARLA, KORA, lidA) (Table 5). Eleven studies had a (meta-)data access infrastructure. Of these, seven offered access without registration, three allowed registration by allowing users to sign up or to send a request per email, and one had no registration option (Fig. 2).

#### Public availability by metadata field

**Fig. 3:**
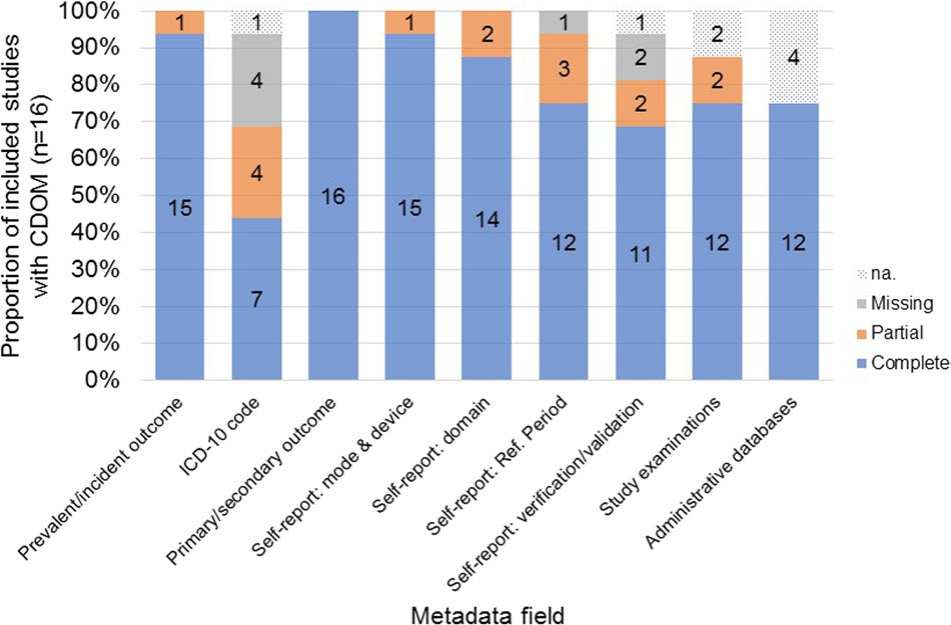
Proportion and number of included studies with complete, partial, and missing publicly available chronic disease outcome metadata, by metadata field (total n = 16). na, does not apply (not part of study design). Metadata considered complete if all aspects of the chronic disease outcome metadata schema (Table 2) are covered for all examined cardiovascular diseases, type 2 diabetes, and cancers.

All publicly available CDOM found was recorded in detail in Supplementary Table 2. Table 6 summarizes this information and rates completeness of CDOM to examine what kind of outcome metadata are more often publicly available or more often missing. A score was applied within each study to evaluate public availability of each metadata field (see evaluation scheme in Table 4: “3”, *complete for all outcomes*; “2”, *complete for some outcomes*; “1”, *partial*; “0“, *missing/no metadata*). Based on these scores, *ICD-10 code* was the field that was more often missing, with a median score of 2. All other metadata fields were more often publicly available, with a median score of 3. Similarly, Fig. 3 reflects the lower availability of information on whether codes of the International Classification of Diseases, Tenth Revision (ICD-10) were used, followed by the fields *self-report: reference period* and *self-report: verification/validation*. Conversely, data on *prevalent/incident outcome* and *primary/secondary outcome* show the highest proportion of completeness.

### Perceived consistency with FAIR principles and perceived barriers

**Fig. 4:**
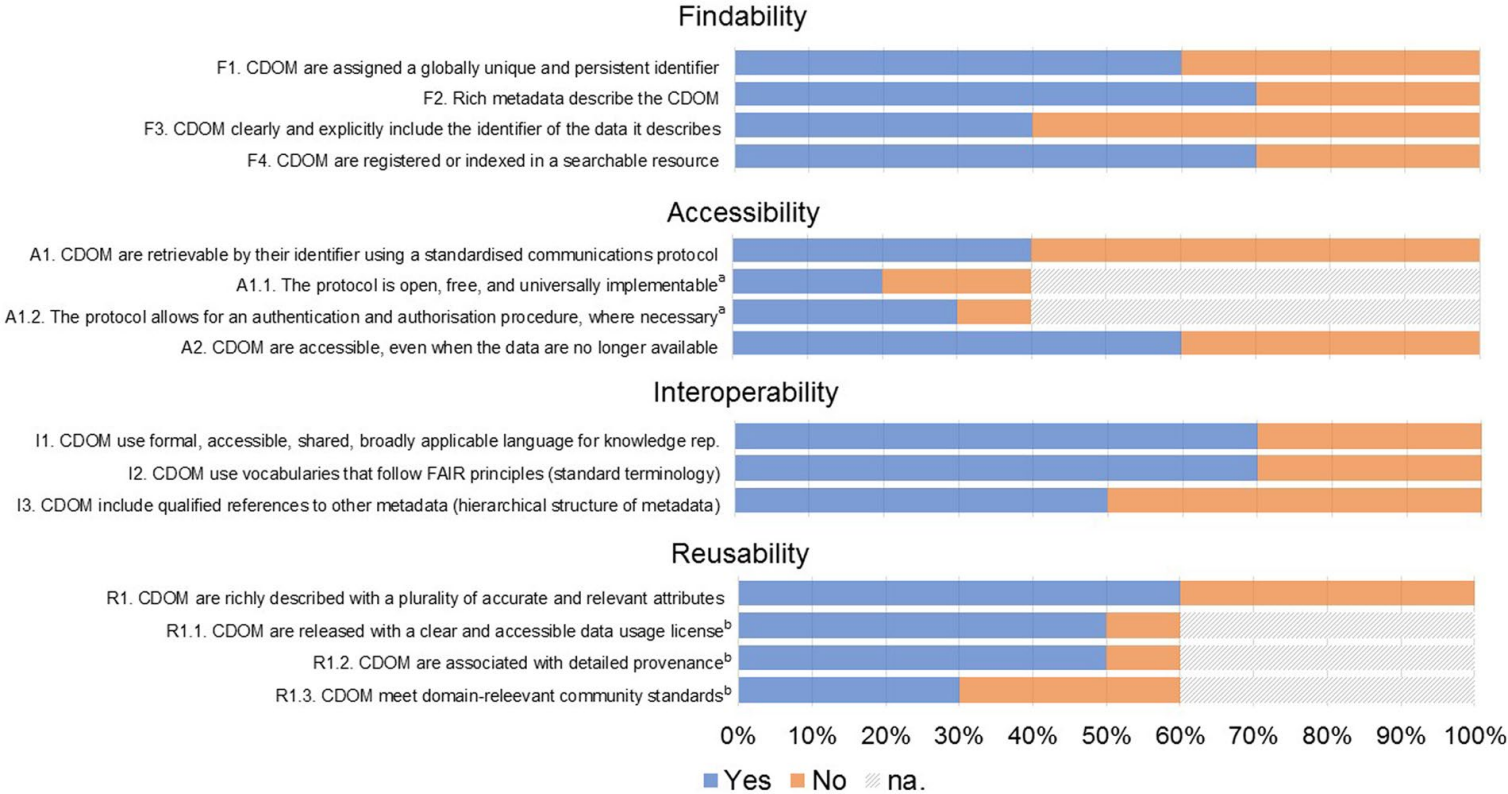
Principal investigators’ (n = 10) perceived consistency of CDOM in their study with FAIR principles. (**a**) Applies only if yes to A1. (**b**) Applies only if yes to R1.

**Fig. 5:**
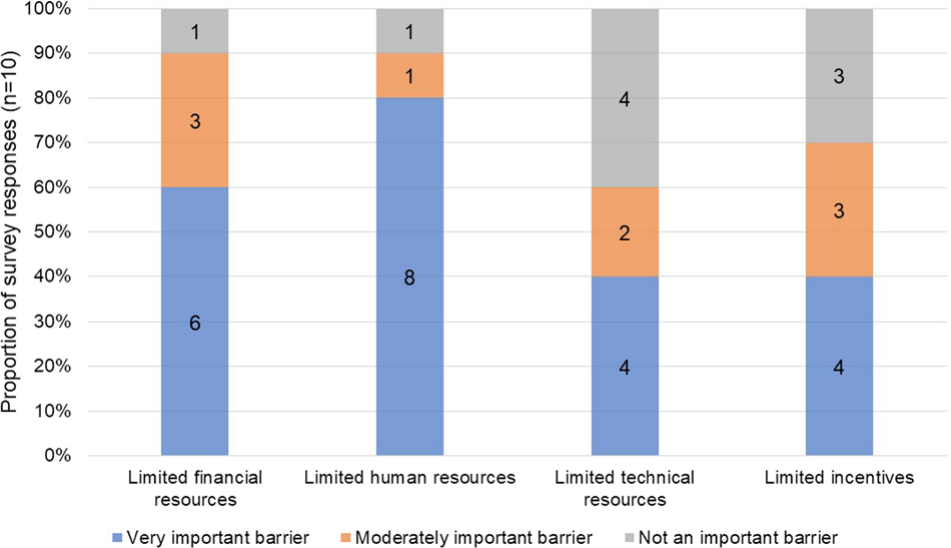
Principal investigators’ (n = 10) perceived barriers to achieve (meta-)data consistency with FAIR principles.

Principal investigators from ten out of the sixteen included studies (one principal investigator by study; N = 10 principal investigators) filled out a survey including the CDOM-adapted checklist of the criteria to meet the FAIR guiding principles (Supplementary Table 3) and shared their perceived main barriers for consistency with the FAIR principles. Principal investigators were prompted to answer always yes/no to each item Perceived consistency of CDOM with FAIR principles ranged from 40% to 70% for findability criteria, from 40% to 60% for accessibility criteria (items A1. and A2.), from 50% to 70% for interoperability criteria, and 60% for reusability criteria (item R1.) (Fig. 4). The main perceived barrier was limited human resources (80% very important barrier, 10% moderately important barrier, 10% not an important barrier), followed by limited financial resources (60% very important barrier, 30% moderately important barrier, 10% not an important barrier) (Fig. 5). Other barriers mentioned by principal investigators were related to unavailability of adequate of harmonization tools, organizational barriers, legal barriers, and limited data quality (Supplementary Table 4).

## Discussion

Based on the proposed CDOM schema, our findings reveal that CDOM from German observational studies are often not fully described in publicly available metadata sources. Among the sixteen included observational studies, six studies had complete publicly available CDOM. The main source of publicly available CDOM were scientific publications and the most frequently missing metadata were whether ICD-10 codes were available, followed by the reference period for the questions from self-reported outcomes and whether and how self-reported outcomes were verified and/or validated.

While CDOM seem to be only partly publicly available, the majority of studies had a (meta-)data access infrastructure accessible without registration, or registration was possible by requesting access. However, about a third of the included studies did not have such infrastructure or it was not publicly accessible. In such cases, data reuse is mostly limited to scientists within specific networks or to those who are already familiar with the studies in question. Rich CDOM that can be found by external parties would substantially assist the scientific community by increasing data interpretability and reusability and thus the value of data and the range of scientific questions on chronic disease risk and progression that could be addressed within and across existing observational studies. Having access to CDOM before the submission of an analysis request would also facilitate study selection and clarify harmonization needs (e.g., for pooled analysis of multiple studies)^11^. For example, knowing whether two studies used different disease classification systems could help the planning of the data harmonization process.

It may not be surprising that our findings suggest the richest source of publicly available CDOM is scientific publications, but it highlights a problem for findability: publications are the traditional way how scientists make research results publicly available. However, these publications usually focus on addressing scientific research questions, rather than on publishing metadata. Although some epidemiological journals also allow the publication of papers on study or cohort profiles^12^, the focus is usually on study design aspects and instruments, rather than on metadata. As a result, metadata are spread across separate documents, often only addressing the necessary information to make sense of the research question(s) addressed in the publication. While finding scientific publications – a time-consuming task that is dependent on search engine and search strategy – may be difficult, finding the metadata within the scientific publications poses another hurdle, as they are not indexed and searchable within the documents^5^. Ideally, CDOM (together with all study metadata) should be centralized (e.g., metadata catalogue on the study’s website) and accessible; and should be linked to publications, data repositories, and other sources of study metadata. By reducing the number of sources repeating the same information and instead linking to a central metadata catalogue or repository, there is a lower risk of inconsistencies (e.g., updating metadata in the primary source but forgetting secondary sources).

There are various reasons why CDOM are often not all publicly available and consistent with FAIR principles. While the concept of FAIR (meta-)data is fairly new^8^, the observational studies included in our evaluation date as far as the 1980s and implementing post hoc classifications of data elements to some standard is difficult and would require considerable resources (i.e., financial, human, and technical) that may not be available. This is in line with our observation that most principal investigators in our survey indicated that limited human resources were the main perceived barrier. Despite these difficulties, there is interest from both more recent, and longer existing German observational studies to improve consistency with the FAIR (meta-)data principles, reflected by their participation in consortia such as NFDI4Health^7^. As the efforts of the included studies to improve adherence to the FAIR principles are ongoing, the findings in this paper reflect the status of CDOM public availability at the time of publication.

Another obstacle for FAIR CDOM is the lack of guidelines or standards for CDOM reporting from observational studies. Our proposed CDOM schema outlines the relevant contextual information that should be included in CDOM reporting to improve interpretability and interoperability. Additionally, it is not clear how FAIRness of CDOM in observational studies should be evaluated. While other FAIR guiding prinples-based evaluation tools have been applied in other fields such as physics and education^13–16^, we considered the checklist we implemented – the FAIR guiding principles^8^ applied to CDOM – to be the most appropriate approach to evaluate the principal investigators’ perception of CDOM FAIRness in their respective observational studies. For this purpose, the breadth of the FAIR guiding principles can allow the principal investigators to consider different implementations of the FAIR principles in their studies. However, comments submitted with the surveys showed that some respondents still found some items difficult to evaluate in the context of CDOM in their study. Other scientists have also found the interpretation challenging and state that the principles should serve as guidelines rather than as standards^5^. Existing standards and classifications such as ICD-10^17^, SNOMED CT^18^, and MIABIS^19^ could be used to establish a specific vocabulary to report CDOM guided by the FAIR princpiles. As these standards and classifications were developed for use in a clinical or health care setting (biomedical research in the case of MIABIS) – although ICD-10 is frequently implemented in epidemiological research – they cover only some CDOM fields (e.g., disease classification in ICD-10, SNOMED-CT, some disease domains and reference periods asked for self-reported outcomes in SNOMED-CT, study examinations in SNOMED-CT and MIABIS). However, different standards and classifications may be used to complement each other and improve CDOM interoperability, for example, by using Unified Medical Language System (UMLS)^20^, which supports the use of multiple vocabularies. To achieve a standard approach, agreements on what standards to use for which metadata fields and on a standard CDOM-reporting template are warranted. Maelstrom Research (https://www.maelstrom-research.org/), which was developed to facilitate epidemiological research collaborations, developed a catalogue^21^ displaying some of the relevant metadata fields for chronic diseases (including ICD-10 disease group classifications); however, it remains mostly on study level metadata, missing outcome-specific metadata. The here proposed CDOM schema offers a blueprint for a more comprehensive metadata model. Resulting comparable contextual information across studies could then be integrated into a common framework such as the ISA-framework in metadata repositories (improved interoperability)^22^.

Our findings should be interpreted in consideration of the study’s strengths and limitations. While there are no guidelines for CDOM reporting in observational studies, we developed a metadata schema for chronic diseases within a large consortium with many participating large German observational studies. We also identify the status regarding public availability of CDOM among German observational studies, contributing knowledge that can be used to target gaps in CDOM findability and accessibility and improve external collaborations in the scientific community. Some limitations of our study include that public availability was conditional on finding the CDOM based on our search criteria; however, the risk of missing important publicly available CDOM was mitigated by requesting feedback from principal investigators about additional internet-available CDOM. Finally, we cannot generalize about the current status of public available CDOM across all observational studies, as all the studies included were from Germany and had already expressed an interest in FAIR data by joining the NFDI4Health consortium; however, most large observational studies conducted in Germany were included.

In summary, CDOM from many population-based observational studies in Germany are not completely publicly available. Those CDOM that are available stem mostly from scientific publications. As studies do not rely on single papers to publish CDOM, findability of these data is limited. There is a need to shift publicly available CDOM from scientific publications to publicly accessible platforms such as easily findable (e.g., visible on the study’s website and linked elsewhere) metadata catalogues (indexed and searchable), where centralization would support data management efforts and completeness of information. This shift requires the availability of the necessary resources for running these platforms, gathering of necessary information, as well as continuous management to keep this information up-to-date on the study level. Furthermore, guidelines or a common approach for how to achieve FAIR CDOM and how to make them publicly available is warranted; for example, a standardised approach to providing data dictionaries and how CDOM are displayed within them. Our findings provide valuable information for the German scientific community and may help justify and impulse efforts to make CDOM fully available in consolidated metadata platforms.

## Methods

### Study selection

This study was conducted within the framework of NFDI4Health. In 2018, the German ministry for education and research (BMBF) and state governments commissioned the German Research Foundation (DFG) to establish a National Research Data Infrastructure (NFDI); in 2019, the DFG launched a first call to form consortia that aim to improve management, accessibility, storage, and sustainability of scientific and research data in all areas of science^23^. NFDI4Health was one of the consortia that successfully applied to the first DFG call, and was selected to be funded for 5 years, starting in 2020^24^. A total of 15 observational studies participated in the funding application for NFDI4Health (i.e., co-applicant studies). NFDI4Health initiated several community workshops to invite potential partners and users from the scientific community to participate in the consortium. Based on this activity, 11 additional observational studies have submitted letters of commitment to participate in the consortium (i.e., participating studies)^24^.

For the current analysis, we selected studies meeting the following inclusion criteria: 1) observational, population-based co-applicant or participating study in NFDI4Health; and 2) collecting information on cardiovascular diseases, cancer, and/or type 2 diabetes mellitus.

### Chronic disease outcome metadata (CDOM) schema

We developed a list of relevant contextual information about chronic disease outcomes for interpretation and reuse of data pertaining to the collection of chronic disease assessment-related information from observational, population-based studies participating in NFDI4Health. A final list of CDOM – a metadata schema specific to chronic disease ascertainment in epidemiological studies (Table 2) – includes general information about the outcome collected (i.e., prevalent or incident case, specific disease name/classification code, primary or secondary outcome) and the assessment method or data source (i.e., from self-report, from study examinations, from administrative databases), with additional levels of detail pertaining to the assessment method. Data pertaining to these metadata fields were searched for each of the eligible studies.

### Sources of chronic disease outcome metadata (CDOM)

Based on an adaptation of previously defined sources contributing to (meta-)data discoverability^25^, the following sources were considered to provide CDOM from epidemiological studies: 1) scientific publications, 2) study websites, 3) study registry databases, and 4) data documents. Table 3 lists these sources in detail. Completeness of published CDOM for all eligible studies was evaluated based on screening of these four metadata sources. Databases used for searching scientific publications were PubMed and Google Scholar, without language restriction. All other sources of metadata were searched using Google including the following predefined keywords: study name, German city/region of the study, and other metadata-source describing keywords. Study/trial registries were searched additionally within websites of the following study registry databases: DRKS (German Clinical Trials Register, https://www.drks.de/), clinicaltrials.gov (https://clinicaltrials.gov/), ISRCTN (International Standard Randomised Controlled Trial Number, https://www.isrctn.com/), Maelstrom Research (https://www.maelstrom-research.org/), re3data.org (https://www.re3data.org/), ICTRP (International Clinical Trials Registry Platform, https://trialsearch.who.int/), euCanSHare (https://eucanshare.bsc.es/platform/), MDM Portal (https://medical-data-models.org/), and German Central Health Study Hub NFDI4Health (https://csh.nfdi4health.de/). Additionally, data documents were searched through the studies’ (meta-)data access infrastructure, if available. Different searches were carried out using terms in English and in German language between January and March 2022. The searches were repeated between August and September 2022 to include newly published CDOM. More details about the search criteria are described in Supplementary Table 1.

### Evaluation of public availability of chronic disease outcome metadata (CDOM)

Public availability of CDOM was evaluated based only on publicly available information from the four aforementioned sources and was defined in terms of findability and accessibility. In a first step, metadata for all included studies were searched by screening in all the predefined metadata sources according to the search criteria detailed in Supplementary Table 1. To be publicly accessible, CDOM had to be both findable and freely accessible on the internet. Availability and accessibility of a (meta-)data access infrastructure was evaluated separately, for which we considered only internet-accessible portals. The existence of such portals was explored within the study website and the search for data documents. After recording all the identified publicly available CDOM by study, principal investigators from all included studies were invited to provide feedback on any missed publicly available CDOM. Any additional CDOM indicated by the principal investigators were added to the results as long as they were available online.

#### Evaluation of publicly available CDOM by study

Public availability of CDOM was evaluated overall for each study, and was considered to be *complete* if a detailed list of all the outcomes of interest that were collected in a study was publicly available and data on all the metadata fields listed in Table 2 was available for each corresponding chronic disease outcome. If data were complete for some outcomes only, published CDOM was considered to be *complete for some outcomes*. If only some of the outcome metadata fields could be filled for one or more chronic disease outcomes, published CDOM was considered to be *partial*. If no metadata fields could be filled based on publicly available information, published CDOM was considered to be *missing*. Table 4 details this evaluation scheme.

#### Evaluation of publicly available CDOM by metadata source and by metadata field

Publicly available CDOM was also recorded in more detail, distinguishing what kind of metadata were found in what source. Based on this information, we calculated a score summarizing public availability of CDOM across all included studies for each metadata field to examine what kind of outcome metadata are more often publicly available or more often missing. Separately for each study and source of metadata, the following rating scheme was used to evaluate each metadata field: “3”, *complete for all outcomes*; “2”, *complete for some outcomes*; “1”, *partial*; “0“, *missing/no metadata* (see Table 4). A score of 1 instead of 2 was given when some details about the metadata field were missing, e.g., if there was an indication that a study collected both prevalent as well as incident outcome data, but only a list of the prevalent outcomes was found (i.e., information about this metadata field was partial). This rating was applied to each outcome metadata field found in each metadata source. As the metadata sources *study website* and *study/trial registries* may serve both as direct sources (i.e., embedded metadata) and indirect sources (i.e., links and references), we evaluated them both as direct sources only and as direct plus indirect sources of metadata. For the overall rating, the highest metadata field score across metadata sources within each study made up the overall rating for a metadata field, which was then used to compute the median score per metadata field (range 0–3). For instance, if a study obtained a “3” for the metadata field “prevalent or incident outcome” based on data documents, but obtained a “2” based on the other metadata sources, the overall score for “prevalent or incident outcome” would be the highest score, i.e., “3” and it would be considered as *complete for all outcomes*.

### Perceived consistency with FAIR principles by the Principal Investigators

Perceived consistency of CDOM with FAIR principles by the principal investigators was assessed based on the previously published criteria for each of the FAIR guiding principles^8^ with regard to CDOM (see Supplementary Table 3). These criteria were circulated as a checklist to the principal investigators of each of the included studies (one principal investigator representing one study), who returned the complete templates for their respective study (see Supplementary Fig. 1). For each criterion, principal investigators had the option of writing a comment, e.g., to express lack of clarity or to provide a more specific answer. Additionally, responders were also asked to provide feedback on their perceived barriers to achieve FAIR (meta-)data for their respective study. The following potential barriers were rated as “very important barrier”, “moderately important barrier” or “not an important barrier”: limited financial resources, limited human resources, limited technical resources, limited incentives. Additional barriers could be entered as free text and were rated in the same way.

### Supplementary information

Supplementary Information

## Data Availability

Data evaluated in this article consists of publicly available metadata; all relevant data are included in the article or uploaded as online supplementary information.
