# Supplementary material for: Chronic disease outcome metadata from German observational studies – public availability and FAIR principles
Source: Sci Data. 2023 Dec 5;10:868. doi: 10.1038/s41597-023-02726-7 (PMC10698176; doi:10.1038/s41597-023-02726-7)

**Chronic disease outcome metadata from German observational studies – public availability and FAIR principles**

**Carolina Schwedhelm^1*^, Katharina Nimptsch^1^, Wolfgang Ahrens^2,3^, Hans M Hasselhorn^4^, Karl-Heinz Jöckel^5^, Verena Katzke^6^, Alexander Kluttig^7,8^, Birgit Linkohr^9^, Rafael Mikolajczyk^7,8^, Ute Nöthlings^10^, Ines Perrar^10^, Annette Peters^9,11^, Carsten O Schmidt^12^, Börge Schmidt^5^, Matthias B Schulze^13,14^, Andreas Stang^5,15^, Hajo Zeeb^2,16^, Tobias Pischon^1,17,18,19^**

^1^ Max-Delbrueck-Center for Molecular Medicine in the Helmholtz Association (MDC), Molecular Epidemiology Research Group, Berlin, 13125, Germany

^2^ Leibniz Institute for Prevention Research and Epidemiology – BIPS, Bremen, 28359, Germany

^3^ University of Bremen, Faculty of Mathematics and Computer Science, Institute of Statistics, Bremen, 28334, Germany

^4^ University of Wuppertal, Department of Occupational Health Science, Wuppertal, 42119, Germany

^5^ University Hospital of Essen, Institute for Medical Informatics, Biometry and Epidemiology, Essen, 45122, Germany

^6^ German Cancer Research Center (DKFZ), Division of Cancer Epidemiology, Heidelberg, 69120, Germany

^7^ Medical Faculty of the Martin-Luther-University Halle-Wittenberg, Institute of Medical Epidemiology, Biometrics and Informatics, Halle (Saale), 06112, Germany

^8^ German Center for Mental Health, Site Jena-Magdeburg-Halle, 07743, Germany

^9^ Helmholtz Zentrum München German Research Center for Environmental Health, Institute of Epidemiology, Neuherberg, 85764, Germany

^10^ University of Bonn, Nutritional Epidemiology, Institute of Nutrition and Food Sciences, Bonn, 53115, Germany

^11^ Medical Faculty of the Ludwig-Maximilians-Universität München, Institute for Medical Information Processing, Biometry and Epidemiology, Department of Epidemiology, Munich, 81377, Germany

^12^ University Medicine Greifswald, Institute for Community Medicine, Greifswald, 17489, Germany

^13^ German Institute of Human Nutrition Potsdam Rehbruecke, Department of Molecular Epidemiology, Nuthetal, 14558, Germany

^14^ University of Potsdam, Institute of Nutritional Science, Nuthetal, 14558, Germany

^15^ Department of Epidemiology, School of Public Health, Boston University, Boston, MA 02118, USA

^16^ University of Bremen, Faculty 11 - Human and Health Sciences, Bremen, 28359, Germany

^17^ Max-Delbrueck-Center for Molecular Medicine in the Helmholtz Association (MDC), Biobank Technology Platform, Berlin, 13125, Germany

^18^ Berlin Institute of Health at Charité – Universitätsmedizin Berlin, Core Facility Biobank, Berlin, 13125, Germany

^19^ Charité – Universitätsmedizin Berlin, Corporate Member of Freie Universität Berlin and Humboldt-Universität zu Berlin, Berlin, 10117, Germany

[*****carolina.schwedhelm@mdc-berlin.de](mailto:*carolina.schwedhelm@mdc-berlin.de)

TABLE OF CONTENTS:

[Supplementary Table 1: Search criteria by metadata source 2](#_Toc150182934)

[Supplementary Table 2: Detailed table on published chronic disease outcome metadata (by source) 3](#_Toc150182935)

[Supplementary Table 3: The FAIR Guiding Principles, applied to metadata^a^ 21](#_Toc150182936)

[Supplementary Table 4: Other perceived barriers to achieve FAIR (meta-)data 21](#_Toc150182937)

[Supplementary references 22](#_Toc150182938)

[Supplementary Figure 1: Principal investigator survey 30](#_Toc150182939)

# Supplementary Table 1: Search criteria by metadata source

| Metadata source | Search engine or database and search criteria | |
| --- | --- | --- |
|  | English search terms (no language restriction) | Additional search with German terms |
| 1. Scientific publications | Search strategy (PubMed and Google Scholar):  #1 [study name] AND [city in Germany^a^] AND cohort profile OR study design OR protocol  #2 [study name] AND [city in Germany^a^] AND disease AND chronic OR non-communicable  #3 [study name] AND [city in Germany^a^] AND cardiovascular OR heart OR stroke OR myocardial infarction OR hypertension  #4 [study name] AND [city in Germany^a^] AND cancer  #5 [study name] AND [city in Germany^a^] AND diabetes | Search strategy (Google Scholar):  #1 [study name] AND [city in Germany^a^] AND Studiendesign OR *protokoll  #2 [study name] AND [city in Germany^a^] AND chronische Erkrankung* OR Nichtübertragbar*  #3 [study name] AND [city in Germany^a^] AND Kardiovaskulär* OR Herz OR Schlaganfall OR Herzinfarkt OR Hypertonie  #4 [study name] AND [city in Germany^a^] AND Krebs |
| 1. Study website | Search strategy (Google):  #1 [study name] AND [city in Germany^a^]  #2 (#1 AND epidemiological OR observational AND study) | Search strategy (Google^b^):  #1 [study name], [city in Germany^a^] AND Studie  #2 (#1 AND epidemiologisch* OR beobachtung*) |
| 1. Study registry databases | Search strategy directly in the following registries: DRKS, clinicaltrials.gov, ISRCTN, Maelstrom Research, re3data.org, ICTRP, euCanSHare, MDM portal, and Central Search Hub NFDI4Health.  Additional search strategy (Google):  #1 [study name] AND [city in Germany^a^] AND study AND (registry OR repository) | Search strategy (Google^b^):  #1 [study name], [city in Germany^a^] AND Studie AND Daten OR Registry |
| 1. Data documentation | Search strategy: thorough search within study website and (meta-)data access infrastructure (web portal).  Additional search strategy (Google): #1 [study name] AND [city in Germany^a^] AND questionnaire OR interview OR data dictionary OR variables | Search strategy (Google^b^):  #1 [study name] AND [city in Germany^a^] AND Befragung* OR Variable*  #2 [study name] AND [city in Germany^a^] AND Daten nutzen OR Forschungsdaten OR Forschungszentrum OR Datenportal OR beantragen |

^a^ If multicentre, term replaced by “German OR Germany” (“Deutsch*” in German language searches).

^b^ Language settings of search engine changed to German.

# Supplementary Table 2: Detailed table on published chronic disease outcome metadata (by source)

| Study | Source of metadata | General information | | |  | | | | | |
| --- | --- | --- | --- | --- | --- | --- | --- | --- | --- | --- |
|  |  | Prevalent/ incident outcome | ICD-10 available | Primary/ secondary outcome | Self-report | | | | Study examinations | Administrative databases^a^ |
|  |  |  |  |  | Mode & Device | Domain | Reference period | Verification/ ext. validation |  |  |
| CARLA | Scientific publications | Prevalent: CHD, stroke, MI, hypertension, diabetes ^1-4^, angina pectoris, intermittent claudication, cancer ^1,3^, heart failure, ventricular hypertrophy, preserved or reduced ejection fraction ^3,4^;  Incident: diabetes, MI, stroke, HF, hypertension ^3,5^ | For lethal (incident) outcomes ^6^ | CVDs are primary outcomes ^1,4^;  Reduced heart rate variability (RHRV) as primary outcome ^3,4^ | Prevalent: CAPI ^1,3,7^;  Incident: CAPI ^3^ | Prevalent: Diagnosis, symptoms (for angina pectoris and intermittent claudication) ^1^,  medical history and medication ^2,3,6^;  Incident: same as baseline (prevalent) ^7^,  disease/diagnosis, symptoms, medications (for CVDs and diabetes ^5^ | Prevalent: last 7 days (medication) ^2,3,6^, ever and currently (Rose questionnaire for intermittent claudication and angina pectoris) ^3^; same format/questions for medical history as KORA and SHIP ^3^;  Incident: last 7 days (medication) ^3,6^; ever and currently (Rose questionnaire for intermittent claudication and angina pectoris) ^3^; same format/questions for medical history as KORA and SHIP ^3^ | Incident: contacting treating physician ^5^ for diagnosis of heart attack, stroke, cancer, diabetes ^3^ | Details on procedures (prevalent & incident): Arterial blood pressure, ECG (used e.g., to determine prevalent MI), trans-thoracic echocardiogram, ankle-brachial index, venous blood (CHD risk based on cholesterol ratio) ^1-5,7^;  for diabetes: HbA1c, blood glucose ^2,5^ | Death certificate for lethal outcomes ^3,6^ |
|  | Study website | *As* ***direct*** *source of metadata:*  Prevalence and incidence of CVDs ^8,9^;  *As* ***indirect*** *source of metadata (links or references):*  Website ref.Nr ^8^ lists the following publications: ^1^;  Newly launched website (2022) ^10^ links to publications: ^1-3^, and links to data dictionaries (baseline and follow-ups): ^11^ | *As* ***indirect*** *source of metadata (links or references):*  Websites ref.Nr ^8,10^ list the following publications: ^6^;  Newly launched website (2022) ^10^ links to data dictionaries (baseline and follow-ups): ^11^ | *As* ***direct*** *source of metadata:*  Primary: CVDs ^8,9^;  *As* ***indirect*** *source of metadata (links or references):*  Website ref.Nr ^8^ lists the following publications: ^1^;  Newly launched website (2022) ^10^ links to study protocol and cohort profile: ^1,3^ | *As* ***indirect*** *source of metadata (links or references):*  Websites ref.Nr ^8,10^ list the following publications: ^1,7^;  Newly launched website (2022) ^10^ links to study protocol and cohort profile: ^1,3^, and links to data dictionaries (baseline and follow-ups): ^11^ | *As* ***indirect*** *source of metadata (links or references):*  Websites ref.Nr ^8,10^ list the following publications: ^1,6^;  Newly launched website (2022) ^10^ links to publications: ^1-3^, and links to data dictionaries (baseline and follow-ups): ^11^ | *As* ***indirect*** *source of metadata (links or references):*  Websites ref.Nr ^8,10^ list the following publications: ^6^;  Newly launched website (2022) ^10^ links to publications ^1-3^, and links to data dictionaries (baseline and follow-ups): ^11^ | *As* ***indirect*** *source of metadata (links or references):*  Newly launched website (2022) ^10^ links to study protocol and cohort profile: ^1,3^, and links to data dictionaries (baseline and follow-ups) :^11^ | *As* ***indirect*** *source of metadata (links or references):*  Websites ref.Nr ^8,10^ list the following publications: ^1,7^;  Newly launched website (2022) ^10^ links to publications: ^1-3^, and links to data dictionaries (baseline and follow-ups): ^11^ | *As* ***indirect*** *source of metadata (links or references):*  Websites ref.Nr ^8,10^ list the following publications: ^6^;  Newly launched website (2022) ^10^ links to study protocol and cohort profile: ^1,3^ |
|  | Study/trial registries | *As* ***indirect*** *source of metadata (links or references):*  MDM Portal entries related to CARLA link to the following publication: ^2^ | - | - | - | *As* ***indirect*** *source of metadata (links or references):*  MDM Portal entries related to CARLA link to the following publication: ^2^ | *As* ***indirect*** *source of metadata (links or references):*  MDM Portal entries related to CARLA link to the following publication: ^2^ | - | *As* ***direct*** *source of metadata:*  MDM Portal: metadata (some details about procedures) from study examinations (blood pressure, ECG, echocardiography)^12^;  *As* ***indirect*** *source of metadata (links or references):*  MDM Portal entries related to CARLA link to the following publication: ^2^ | - |
|  | Data documents | All outcomes listed as variables (baseline and follow-ups) ^11^ | Prevalent & incident: ICD code variables for cancer and other chronic diseases ^11^ | - | Prevalent & incident: specification by variable in data dictionary ^11^ | Prevalent & incident: specified in variable labels ^11^ | Prevalent & incident: specified in variable labels ^11^ | Derived and verified variables included in data dictionary (baseline and follow-ups) ^11^ | Corresponding variables in data dictionary (baseline and follow-ups) ^11^ | - |
| DEGS1 | Scientific publications | Prevalent: angina pectoris or other CHD, MI, HF, PAD, stroke, hypertension, diabetes mellitus, any type of cancer ^13^;  Incident: diabetes, MI, stroke, hypertension ^5^ | - | Health monitoring. No specific primary/secondary outcomes ^14,15^ | Prevalent: CAPI ^13-16^;  Incident (for GNHIES98 participants): same as baseline (prevalent) ^15^; | Prevalent: Diagnosis, Treatment and medications ^13,15,17,18^;  Incident (for GNHIES98 participants): same as baseline (prevalent) ^15^ | Prevalent: Last 7 days (medications) ^15,17^, ever (diagnosis) ^13,17,18^, last year (presence of previous diseases) ^13^;  Incident (for GNHIES98 participants): same as baseline (prevalent) ^15^ | None ^5^ | Prevalent and incident (for GNHIES98 participants): Blood pressure, heart rate, blood sample ^13,14^; criteria to determine T2D ^17^ | - |
|  | Study website | *As* ***direct*** *source of metadata:*  Prevalent: obesity, allergies, asthma, hypertension, CVD, diabetes, metabolic syndrome, musculoskeletal disorders ^19^;  *As* ***indirect*** *source of metadata (links or references):*  Websites ref.Nr ^19,20^ link to data documents (variable list) ^21^ | - | *As* ***direct*** *source of metadata:*  Health monitoring ^19,20^;  *As* ***indirect*** *source of metadata (links or references):*  Websites ref.Nr ^19,20^ link to the following publications: ^14,15^ | *As* ***direct*** *source of metadata:*  Face-to-face interview (paper/computer-based not specified) ^19^;  *As* ***indirect*** *source of metadata (links or references):*  Websites ref.Nr ^19,20^ link to the following publications: ^14-16^ | *As* ***direct*** *source of metadata:*  Prevalent: disease, diagnosis, treatment and medications ^19^;  *As* ***indirect*** *source of metadata (links or references):*  Websites ref.Nr ^19,20^s link to the following publications: ^15,17,18^, as well as to data documents ^21^ | *As* ***direct*** *source of metadata:*  Prevalent: last 7 days (medications) ^19^;  *As* ***indirect*** *source of metadata (links or references):*  Websites ref.Nr ^19,20^ link to the following publications: ^15,17,18^, as well as to data documents ^21^ | - | *As* ***direct*** *source of metadata:*  Prevalent: blood pressure, heart rate, blood sample ^19^;  *As* ***indirect*** *source of metadata (links or references):*  Websites ref.Nr ^19,20^ link to the following publications: ^14,17^, as well as to data documents ^21^ | *As* ***direct*** *source of metadata:*  Cause of death from death certificate starting 2016 ^19^ |
|  | Study/trial registries | na. | na. | na. | na. | na. | na. | na. | na. | na. |
|  | Data documents | Available from variable list ^21^ (DEGS1 only); incident data missing | - | - | - | Available from variable list ^21^ (DEGS1 only); incident data missing | Available from variable list ^21^ (DEGS1 only); incident data missing | - | Available from variable list ^21^ (DEGS1 only); incident data missing | - |
| DONALD | Scientific publications | Enrolment at 3 months; chronic diseases are incident. All diagnoses get recorded ^22,23^ | All diseases ICD-coded ^22^ | Primary: dietary intake, growth, development, metabolism ^22-30^ | Face-to-face interview (paper/computer-based not specified) ^22^ | Disease (did you have X?), Medications ^22^ | Last visit (disease) ^22^ | - | Blood pressure (procedure explained) ^25,26,29^;  Venous blood sample in adulthood (procedure explained; analysed T2D, CVD, inflammation markers)^23,24,28,30,31^;  Intima media thickness of common carotid artery (procedure explained) ^27^ | na. |
|  | Study website | *As* ***direct*** *source of metadata:*  Enrolment at 3 months; chronic diseases are incident. All diagnoses get recorded ^32^;  *As* ***indirect*** *source of metadata (links or references):*  Website ref.Nr ^32^ lists the following publication: ^22^ | *As* ***indirect*** *source of metadata (links or references):*  Website ref.Nr ^32^ lists the following publication: ^22^ | *As* ***direct*** *source of metadata:*  Primary: dietary intake, growth, development, metabolism ^32^;  *As* ***indirect*** *source of metadata (links or references):*  Website ref.Nr ^32^ lists the following publications: ^22,30^ | *As* ***indirect*** *source of metadata (links or references):*  Website ref.Nr ^32^ lists the following publication: ^22^ | *As* ***direct*** *source of metadata:*  Disease (did you have X?) ^32^;  *As* ***indirect*** *source of metadata (links or references):*  Website ref.Nr ^32^ lists the following publication: ^22^ | *As* ***direct*** *source of metadata:*  Last visit (disease) ^32^;  *As* ***indirect*** *source of metadata (links or references):*  Website ref.Nr ^32^ lists the following publication: ^22^ | - | *As* ***direct*** *source of metadata:*  Blood pressure (details not provided), intima media thickness of common carotid artery, blood samples (glucose among measures) ^32^;  *As* ***indirect*** *source of metadata (links or references):*  Website ref.Nr ^32^ lists the following publications: ^22,30,31^ | na. |
|  | Study/trial registries | *As* ***direct*** *source of metadata:*  Only healthy infants are included (outcomes are incident): cardiometabolic risk markers in adulthood, carotid intima media thickness, among other (non-CVD, diabetes or cancer-related) ^33-35^; blood pressure, chronic diseases ^36^;  *As* ***indirect*** *source of metadata (links or references):*  Registries ^34-36^ link to study website ^32^; registries ^35,36^ link to publication ^22^ | *As* ***indirect*** *source of metadata (links or references):*  Registries ^35,36^ link to publication ^22^ | *As* ***direct*** *source of metadata:*  No primary/secondary endpoints (longitudinal study during growth)^33,34^; dietary intake, growth, development, metabolism ^35^;  *As* ***indirect*** *source of metadata (links or references):*  Registries ^34-36^ link to study website ^32^; registries ^35,36^ link to publication ^22^ | *As* ***direct*** *source of metadata:*  Face-to-face interview, paper-based ^36^;  *As* ***indirect*** *source of metadata (links or references):*  Registries ^35,36^ link to publication ^22^ | *As* ***indirect*** *source of metadata (links or references):*  Registries ^34-36^ link to study website ^32^; registries ^35,36^ link to publication ^22^ | *As* ***indirect*** *source of metadata (links or references):*  Registries ^34-36^ link to study website ^32^; registries ^35,36^ link to publication ^22^ | - | *As* ***direct*** *source of metadata:*  Ultrasound of carotid intima media from age 12 years, blood pressure from age 6 years ^33,34^; blood drawn in adulthood ^34-36^;  *As* ***indirect*** *source of metadata (links or references):*  Registries ^34-36^ link to study website ^32^; registries ^35,36^ link to publication ^22^ | na. |
|  | Data documents | na. | na. | na. | na. | na. | na. | na. | na. | na. |
| EPIC-Heidelberg | Scientific publications | Incident and prevalent: malignant tumours, benign tumours, MI, CHD/angina pectoris, stroke, cerebral circulatory disorders, diabetes, hypertension ^37^ | Incident outcomes (CVDs, cancer) ^38-44^ | Primary: cancer, later expanded to other diseases like CVDs (chronic diseases in general are primary outcomes now) ^45^; | Prevalent: CAPI ^37^;  Incident (passive follow-up): Self-completed, paper-based ^37^ | Prevalent and incident (active follow-up): diagnosis ^45,46^, medication ^45^ | Incident: (active follow-up): since last examination (diagnosis), 4 weeks (medication) ^45^ | Incident (active follow-up): verified with hospitalization records, and cancer and pathology registries ^37,41,46,47^; review of medical records by study doctor ^40-43,46,48^; contacting treating physician ^37^ | Blood pressure, blood samples, ^42,45^; pulse rate ^45^ | Incident cancers (passive follow-up): record linkage to cancer registries ^37,41^;  Prevalent and incident: cancer registry, death certificate, clinics and physicians used to find all cases ^37^;  Death certificates ^37,38,41,43,46^ |
|  | Study website | *As* ***direct*** *source of metadata:*  Incident cancers and other chronic diseases ^46,49^;  *As* ***indirect*** *source of metadata (links or references):*  Website ref.Nr ^49^ lists the following publication: ^37^ | - | *As* ***indirect*** *source of metadata (links or references):*  Website ref.Nr ^49^ lists the following publication: ^45^ | *As* ***indirect*** *source of metadata (links or references):*  Website ref.Nr ^49^ lists the following publication: ^37^ | *As* ***direct*** *source of metadata:*  Prevalent (baseline): medical history, medications;  Incident (follow-up): new diseases ^49^  *As* ***indirect*** *source of metadata (links or references):*  Website ref.Nr ^49^ lists the following publication: ^45^ | *As* ***direct*** *source of metadata:*  Incident (follow-up): since last examination (disease) ^49^  *As* ***indirect*** *source of metadata (links or references):*  Website ref.Nr ^49^ list publications ^45^ | *As* ***direct*** *source of metadata:*  Incident: selected cases are verified by contacting treating physicians and hospitals/clinics ^49^  *As* ***indirect*** *source of metadata (links or references):*  Website ref.Nr ^49^ lists the following publications: ^37,48^ | *As* ***direct*** *source of metadata:*  Prevalent (baseline): Blood samples, blood pressure measurements (no details provided) ^49^  *As* ***indirect*** *source of metadata (links or references):*  Website ref.Nr ^49^ lists the following publication: ^45^ | *As* ***direct*** *source of metadata:*  Cancer cases: health insurance records, cancer registries (EPIC IARC website ^50^);  *As* ***indirect*** *source of metadata (links or references):*  Website ref.Nr ^49^ lists the following publication: ^37^ |
|  | Study/trial registries | na. | na. | na. | na. | na. | na. | na. | na. | na. |
|  | Data documents | na. | na. | na. | na. | na. | na. | na. | na. | na. |
| EPIC-Potsdam | Scientific publications | Prevalent: type 2 diabetes, cancer, CVDs ^51-55^;  Incident: malignant tumours, benign tumours, MI, CHD/angina pectoris, stroke, cerebral circulatory disorders, diabetes, hypertension ^37^; | Prevalent: no information found;  Incident outcomes ^51,53-56^ | Primary: incidence of cancer, later expanded to other diseases like CVDs (chronic diseases in general are primary outcomes now) ^45^; | Prevalent: CAPI ^37^;  Incident (active follow-up): Self-completed, paper-based ^37,52^; | Prevalent and incident (active follow-up): diagnosis, medication, treatment, dietary change ^45,52-55,57^ | Incident: (active follow-up): since last examination (diagnosis) ^45^, last 4 weeks (medication) ^45,52^, current (medication and/or treatment) ^57^ | Incident cancers (active follow-up): verified with cancer registries ^37,55^;  Incident non-cancer diseases: contacting treating physician ^37,51,52,54,55,57^;  Incident outcomes: medical records from treating physicians/cancer registries/death certificates ^53,56^ | Blood pressure ^45,52-54,58^;  blood samples ^45,54,57^;  pulse rate ^45^; | Incident cancers (passive follow-up): record linkage to cancer registries ^37,59^;  Prevalent and incident: cancer registry, death certificate, clinics and physicians used to find all cases ^37^;  Death certificates ^37,54^ |
|  | Study website | *As* ***direct*** *source of metadata:*  Incidence of cancer and other chronic diseases like type 2 diabetes ^60^  *As* ***indirect*** *source of metadata (links or references):*  DKFZ website for EPIC-Heidelberg (^49^) includes list of publications relevant to EPIC-Potsdam ^37^ |  | *As* ***indirect*** *source of metadata (links or references):*  DKFZ website for EPIC-Heidelberg (^49^) includes list of publications relevant to EPIC-Potsdam ^45^ | *As* ***direct*** *source of metadata:*  Incident (follow-up): self-completed, paper-based ^60^;  *As* ***indirect*** *source of metadata (links or references):*  DKFZ website for EPIC-Heidelberg (^49^) includes list of publications relevant to EPIC-Potsdam ^37^ | *As* ***direct*** *source of metadata:*  Incident: disease ^60^;  *As* ***indirect*** *source of metadata (links or references):*  DKFZ website for EPIC-Heidelberg (^49^) includes list of publications relevant to EPIC-Potsdam ^45^ | *As* ***direct*** *source of metadata:*  Incident (follow-up): since last examination ^60^;  *As* ***indirect*** *source of metadata (links or references):*  DKFZ website for EPIC-Heidelberg (^49^) includes list of publications relevant to EPIC-Potsdam ^45^ | *As* ***direct*** *source of metadata:*  Incident cases verified through medical documents, registers or physicians ^60^;  *As* ***indirect*** *source of metadata (links or references):*  DKFZ website for EPIC-Heidelberg (^49^) includes list of publications relevant to EPIC-Potsdam ^37^ | *As* ***direct*** *source of metadata:*  Incident (baseline): blood pressure, blood samples ^60^;  *As* ***indirect*** *source of metadata (links or references):*  DKFZ website for EPIC-Heidelberg (^49^) includes list of publications relevant to EPIC-Potsdam ^45^ | *As* ***direct*** *source of metadata:*  Cancer cases: health insurance records, cancer registries (EPIC IARC website ^50^);    *As* ***indirect*** *source of metadata (links or references):*  DKFZ website for EPIC-Heidelberg (^49^) includes list of publications relevant to EPIC-Potsdam ^37^ |
|  | Study/trial registries | *As* ***direct*** *source of metadata:*  Development and progression (incidence) of chronic diseases, including T2D, MI, stroke, colorectal cancer  ^33,34^ | *As* ***direct*** *source of metadata:*  ICD-10 codes provided for health conditions studied (seems to apply to incident outcomes, unclear if prevalent outcomes are also ICD-coded) ^33,34^ | *As* ***direct*** *source of metadata:*  Primary: T2D, cancer, CVD, colitis, stroke, MI, HF, hypertension; Secondary: diabetes-associated complications ^33,34^; cancer and other chronic diseases ^35^;  *As* ***indirect*** *source of metadata (links or references):*  Registries ^35,36^ list the following publication: ^45^ | *As* ***direct*** *source of metadata:*  Prevalent: face-to-face (paper/computer-based no specified); Incident: self-completed, paper-based ^33,34^ | *As* ***direct*** *source of metadata:*  Incidence: diagnosis, medication/treatment, dietary changes due to disease ^33,34^;  *As* ***indirect*** *source of metadata (links or references):*  Registries ^35,36^ list the following publication: ^45^ | *As* ***indirect*** *source of metadata (links or references):*  Registries ^35,36^ list the following publication: ^45^ | *As* ***direct*** *source of metadata:*  Incident cases verified by forms filled by treating physician ^33,34^ | *As* ***direct*** *source of metadata:*  Blood pressure, blood sampling ^34^;  *As* ***indirect*** *source of metadata (links or references):*  Registries ^35,36^ list the following publication: ^45^ | *As* ***direct*** *source of metadata:*  Death certificates, tumour centres, clinical records linkage ^33,34^ |
|  | Data documents | na. | na. | na. | na. | na. | na. | na. | na. | na. |
| GEDA 2009, 2010, 2012, 2014/2015, 2019/2020, 2021 | Scientific publications | Prevalent: hypertension, diabetes, stroke, MI, angina pectoris, CHD, chronic HF ^61-64^, cancer ^61-63^;  Incident: na. | na. | Health monitoring, chronic diseases in general. No specific primary/secondary outcomes ^61-64^ | Prevalent: CATI ^61,62,64^; GEDA 2014/2015 was self-completed, paper- or web-based ^63^;  Incident: na. | Prevalent: diagnosis ^61-63^, disease (have you had) ^64^;  Incident: na. | Prevalent: ever and/or 1 year ^61-64^;  Incident: na. | na. | na. | na. |
|  | Study website | *As* ***direct*** *source of metadata:*  Prevalent: chronic diseases (no detailed specification); Incident: na. ^65^;  *As* ***indirect*** *source of metadata (links or references):*  Website ref.Nr ^66^ lists the following publications:  ^63,64^ | na. | *As* ***direct*** *source of metadata:*  Health monitoring, chronic diseases in general. No specific primary/secondary outcomes ^65,66^;  *As* ***indirect*** *source of metadata (links or references):*  Website ref.Nr ^66^ lists the following publications: ^63,64^ | *As* ***direct*** *source of metadata:*  CATI ^65,66^;  *As* ***indirect*** *source of metadata (links or references):*  Website ref.Nr ^66^ lists the following publications: ^63,64^ | *As* ***indirect*** *source of metadata (links or references):*  Website ref.Nr ^66^ lists the following publications: ^63,64^ | *As* ***indirect*** *source of metadata (links or references):*  Website ref.Nr ^66^ lists the following publications: ^63,64^ | na. | na. | na. |
|  | Study/trial registries | na. | na. | na. | na. | na. | na. | na. | na. | na. |
|  | Data documents | Available from variable lists up to GEDA 2019/2020 ^21^, questionnaire in supplement ^63,64^ | na. | - | - | Available from variable lists up to GEDA 2019/2020 ^21^, questionnaire in supplement ^63,64^ | Available from variable lists up to GEDA 2019/2020 ^21^, questionnaire in supplement ^63,64^ | na. | na. | na. |
| GHS | Scientific publications | Prevalent: MI, incident stroke, diabetes, HF, atrial fibrillation, cancer ^67^;  Incident: MI, incident stroke, diabetes, HF, atrial fibrillation, cancer, cardiovascular mortality  ^67^ | Incident CVDs ^68-70^ | Primary: CVDs (and cardiovascular risk factors) ^69-73^  Primary: incident MI, cardiovascular mortality;  Secondary: incident stroke, diabetes, HF, atrial fibrillation, mortality from these outcomes;  Tertiary: cancer  ^67^ | Prevalent: CAPI ^67,74-76^;  Incident: CATI ^67^ | Diagnosis, treatment, symptoms, medication ^67,77^; history, type, date of diagnosis (cancer) ^71^ | Since the examination in the study centre (2.5 years) ^67^; current (medication)^74^ | Incident: endpoint committee evaluates treatment documentation provided by participant ^67^ (participants brought their medical records, if available ^71,74^) | Blood pressure, heart rate, ECG, echocardiography, ankle-brachial index ^67^; endothelial function determined by flow-mediated dilation and peripheral arterial tonometry ^78^; blood samples (incl. HbA1c, fasting glucose, non-fasting glucose) ^67,71^; carotid intima media thickness ^79^;  Angiography, MRT, heart enzymes ^5^;  Criteria (based on study examinations) for determination of prevalent diabetes, prediabetes, hypertension, atrial fibrillation, dyslipidaemia, arterial stiffness provided ^69,71,76,79-82^;  Venous insufficiency assessed according to CEAP classification scheme (in standardized clinical examination) ^75^ | Death certificates ^67,69,72,75^; health records ^70^ |
|  | Study website | - | - | *As* ***direct*** *source of metadata:*  Primary: cardiovascular health ^83,84^;  Secondary: cancer, eye diseases, immune system, metabolism and psyche ^83,84^ | *As* ***direct*** *source of metadata:*  CAPI (baseline and after 5 years), CATI (after 2.5 and 7.5 years) ^84^ | *As* ***direct*** *source of metadata:*  Medications ^83^ | *As* ***direct*** *source of metadata:*  Last month (medications) ^83^ | - | *As* ***direct*** *source of metadata:*  Ultrasound of the heart/echocardiography, ultrasound of the carotid artery/sonography of the carotid bifurcation, measurement of vascular stiffness and pulse wave analysis, peripheral occlusion pressure measurement, resting blood pressure and resting heaeret rate, ECG, blood sampling (information participant-oriented, no details on procedures) ^83^ | - |
|  | Study/trial registries | - | - | *As* ***direct*** *source of metadata:*  Primary: CVDs; Secondary: cancer, eye diseases, diseases of the immune system, metabolic and mental diseases ^85^;  *As* ***indirect*** *source of metadata (links or references):*  Synchros cohort repository ^85^ links to study website ^83^ | - | *As* ***indirect*** *source of metadata (links or references):*  Synchros cohort repository ^85^ links to study website ^83^ | *As* ***indirect*** *source of metadata (links or references):*  Synchros cohort repository ^85^ links to study website ^83^ | - | *As* ***indirect*** *source of metadata (links or references):*  Synchros cohort repository ^85^ links to study website ^83^ | - |
|  | Data documents | na. | na. | na. | na. | na. | na. | na. | na. | na. |
| GNHIES98 (BGS98) | Scientific publications | Prevalent: MI, stroke (only non-lethal), diabetes ^86^;  Incident: na. | - | Health monitoring, chronic diseases in general. No specific primary/secondary outcomes ^86^ | Prevalent: self-completed (paper-based) and face-to-face (paper/computer-based not specified) ^86^;  Incident: na. | Prevalent MI, stroke, diabetes: disease (self- completed); disease/diagnosis, treatment (face-to-face) ^86^;  Incident: na. | Prevalent: ever (MI, stroke, diabetes) ^86^;  Incident: na. | Prevalent: comparison self-completed questionnaire vs. interview with study doctor (MI, stroke, diabetes) ^86^;  Incident: na. | Prevalent diabetes: serum glucose, HbA1c, fructosamine, urine glucose, if 2 of these criteria were elevated (cut-offs provided), then it was considered diabetes ^86^;  Prevalent hypertension: blood pressure, details provided ^86^. | na. |
|  | Study website | *As* ***indirect*** *source of metadata (links or references):*  Website ref.Nr ^87^ lists the following set of publications: ^86^; it also links to data documents ^21^ | - | *As* ***direct*** *source of metadata:*  Health monitoring, chronic diseases in general. No specific primary/secondary outcomes ^87^;  *As* ***indirect*** *source of metadata (links or references):*  Website ref.Nr ^87^ lists the following set of publications: ^86^ | *As* ***indirect*** *source of metadata (links or references):*  Website ref.Nr ^87^ lists the following set of publications: ^86^ | *As* ***indirect*** *source of metadata (links or references):*  Website ref.Nr ^87^ lists the following set of publications: ^86^; it also links to data documents ^21^ | *As* ***indirect*** *source of metadata (links or references):*  Website ref.Nr ^87^ lists the following set of publications: ^86^; it also links to data documents ^21^ | *As* ***indirect*** *source of metadata (links or references):*  Website ref.Nr ^87^ lists the following set of publications: ^86^ | *As* ***indirect*** *source of metadata (links or references):*  Website ref.Nr ^87^ lists the following set of publications: ^86^; it also links to data documents ^21^ | na. |
|  | Study/trial registries | na. | na. | na. | na. | na. | na. | na. | na. | na. |
|  | Data documents | Available from variable list ^21^ | - | - | - | Disease (from variable list ^21^) | Ever (from variable list ^21^) | - | Measured blood pressure (from variable list, details not provided ^21^) | na. |
| HCHS | Scientific publications | Prevalent and incident: >30 major chronic diseases (incl. CVDs, T2D, cancer) ^88^ | - | Primary: > 30 major chronic diseases (incl. CVDs, T2D, cancer) ^88^ | Prevalent and incident: self-completed, paper-based ^88^ | Prevalent: medical history, medication ^89^ | - | Incident: diseases validated through health insurance data; cancer through cancer registries ^88^ | Prevalent CVDs: blood pressure, echocardiography, ultrasound of abdominal aorta, peripheral venous system and carotid artery, ABI, ECG ^88^;  Prevalent diabetes: blood glucose and HbA1c ^88^, cut off for fasting and non-fasting glucose provided ^90^;  Prevalent carotid atherosclerosis: measurement of intima media thickness using sonography, cut off provided ^90^;  Prevalent hypertension: blood pressure measurements, details and cut off provided ^90^;  Prevalent hypercholesterolemia: LDL-cholesterol cut off provided ^90^;  Prevalent atrial fibrillation: based on ECG, interpreted by 2 experienced cardiologists ^91^ | Death certificates ^88^ |
|  | Study website | *As* ***indirect*** *source of metadata (links or references):*  Website ref.Nr ^92^ links to the following publication: ^88^ | - | *As* ***indirect*** *source of metadata (links or references):*  Website ref.Nr ^92^ links to the following publication: ^88^ | *As* ***indirect*** *source of metadata (links or references):*  Website ref.Nr ^92^ links to the following publication: ^88^ | *As* ***indirect*** *source of metadata (links or references):*  Website ref.Nr ^92^ links to the following publication: ^89^ | - | *As* ***indirect*** *source of metadata (links or references):*  Website ref.Nr ^92^ links to the following publication: ^88^ | *As* ***indirect*** *source of metadata (links or references):*  Website ref.Nr ^92^ links to the following publication: ^88,89,91^ | *As* ***indirect*** *source of metadata (links or references):*  Website ref.Nr ^92^ links to the following publication: ^88^ |
|  | Study/trial registries | *As* ***direct*** *source of metadata:*  Cancer, coronary heart disease, stroke, vascular diseases, among others (no specification prevalent/incident) ^33^;  Prevalent (baseline): list of CVD-related diseases ^93^;  *As* ***indirect*** *source of metadata (links or references):*  Registries ref.Nr ^85,93,94^ link to website ^92^ and the following publication: ^88^ | - | *As* ***direct*** *source of metadata:*  Primary: CHD, stroke, dementia, cancer, vascular diseases, periodontal diseases, ocular diseases, respiratory disease, obesity ^95^; major chronic diseases ^85^;  *As* ***indirect*** *source of metadata (links or references):*  Registries ref.Nr ^85,93,94^ link to website ^92^ and the following publication: ^88^ | *As* ***direct*** *source of metadata:*  Incident CAD, atrial fibrillation, HF, stroke, cancer: self-completed (paper/computer-based not specified) ^95^;  *As* ***indirect*** *source of metadata (links or references):*  Registries ref.Nr ^85,93,94^ link to website ^92^ and the following publication: ^88^ | *As* ***indirect*** *source of metadata (links or references):*  Registries ref.Nr ^93-95^ link to website ^92^ | - | *As* ***indirect*** *source of metadata (links or references):*  Registries ref.Nr ^85,93,94^ link to website ^92^ and the following publication: ^88^ | *As* ***direct*** *source of metadata:*  Serum, plasma, MRI (heart or brain) in subsample at risk ^95^;  Prevalent (baseline): ECG, blood samples, imaging of heart, carotid ^93^;  *As* ***indirect*** *source of metadata (links or references):*  Registries ref.Nr ^85,93,94^ link to website ^92^ and the following publication: ^88^ | *As* ***indirect*** *source of metadata (links or references):*  Registries ref.Nr ^85,93,94^ link to website ^92^ and the following publication: ^88^ |
|  | Data documents | na. | na. | na. | na. | na. | na. | na. | na. | na. |
| HNRS | Scientific publications | Prevalent: coronary artery disease, tumours ^96^; T2D, hypertension ^97^  Incident: MI, stroke, T2D, HF, hypertension ^5^; incident PAD ^98^ | Prevalent and incident tumours: ICD-10 derived ^96^;  Cardiovascular mortality ICD-10 classified ^99,100^;  Coronary events ICD-10 classified ^101^ | Primary: coronary arteriosclerosis ^98,102^;  Primary: MI and cardiac death ^97,103^; Secondary: overall mortality, cerebrovascular events, other CVDs ^97^ | Prevalent PAD: CAPI ^98^;  All prevalent and incident (5 yr and 10 yr FU): CAPI ^97^;  Incident (yearly questionnaire): self-completed, paper-based ^97,103^ | Prevalent PAD: diagnosis ^98^;  Prevalent diabetes: diagnosis, medication ^104^;  Incident CVDs, diabetes: disease/diagnosis, treatment/medication, symptoms ^5^;  Prevalent and incident (5y FU): Disease, medications ^105^ | Prevalent PAD: Ever (Dx) ^98^ | Incident: treating physician ^5^;  Prevalent PAD: not validated ^98^;  Treatment documentation provided by participants (including medical records) reviewed by endpoint committee ^97,99-101^;  Agreement between self-reports and medical records was evaluated ^106^ | Blood pressure, ECG, ABI, EBCT ^102^;  Stress ECG ^96^;  Blood samples (glucose, HbA1c, total cholesterol, HDL-cholesterol), blood pressure (procedure explained), ABI, stress ECG ^97^ | Death certificates ^96,99,100^;  Medical records ^107^ |
|  | Study website | - | - | *As* ***direct*** *source of metadata:*  Primary: CVDs ^108^ | - | - | - | - | - | - |
|  | Study/trial registries | *As* ***indirect*** *source of metadata (links or references):*  German Biobank Registry ^109^ links to publications ^98^ | - | *As* ***direct*** *source of metadata:*  Primary: atherosclerosis, MI, stroke ^109^;  *As* ***indirect*** *source of metadata (links or references):*  German Biobank Registry ^109^ links to publications ^98^ | *As* ***indirect*** *source of metadata (links or references):*  German Biobank Registry ^109^ links to publications ^98^ | *As* ***direct*** *source of metadata:*  Diagnosis, medication, tumour-specific data (size, stage, radiology findings) ^109^;  *As* ***indirect*** *source of metadata (links or references):*  German Biobank Registry ^109^ links to publications ^98^ | *As* ***indirect*** *source of metadata (links or references):*  German Biobank Registry ^109^ links to publications ^98^ | *As* ***indirect*** *source of metadata (links or references):*  German Biobank Registry ^109^ links to publications ^98^ | *As* ***direct*** *source of metadata:*  Prevalent and incident coronary atherosclerosis: electron-beam computed tomography (no details provided) ^109^;  Blood samples, blood pressure, pulse (no details provided) ^109^ | *As* ***direct*** *source of metadata:*  Electronic health databases (death or hospitalization registries) ^109^ |
|  | Data documents | na. | na. | na. | na. | na. | na. | na. | na. | na. |
| IDEFICS/ I.Family | Scientific publications | Prevalent and incident: lifestyle-related health outcomes (e.g., overweight/obesity, metabolic syndrome, hypertension, bone health, mental well-being) ^110^; | All coded according to ICD-10 ^111^ | Primary: non-communicable chronic diseases and disorders ^110^ | CAPI ^110-112^ | Diagnosis, medications, treatment ^111^ | Ever (diagnosis), current (disease still present, medications), +/- 1-month duration (treatment) ^111^ | Measured HbA1c used to verify fasting blood glucose (since fasting status is self-reported) ^111^ | Fasting blood, urine samples, pulse rate, blood pressure, anthropometry, bone ultrasonography ^110-112^;  Several blood and DNA markers incl. serum glucose, insulin, HbA1c, lipids, Vitamin D, CRP, whole genome scan, etc. ^111,112^;  Definitions and cut-offs of hypertension ^113^ and metabolic syndrome based on study examinations ^114^ | Maternity cards and records of routine child visits ^110^ |
|  | Study website | *As* ***direct*** *source of metadata:*  Blood pressure, insulin resistance (no distinction prevalent/incident) ^115^;  *As* ***indirect*** *source of metadata (links or references):*  Websites ref.Nr.^115,116^ link to the following publications: ^110,111^ | *As* ***indirect*** *source of metadata (links or references):*  Websites ref.Nr.^115,116^ link to the following publication: ^111^ | *As* ***indirect*** *source of metadata (links or references):*  Websites ref.Nr.^115,116^ link to the following publications: ^110,111^ | *As* ***indirect*** *source of metadata (links or references):*  Websites ref.Nr.^115,116^ link to the following publications: ^110-112^ | *As* ***indirect*** *source of metadata (links or references):*  Websites ref.Nr.^115,116^ link to the following publication: ^111^ | - | *As* ***indirect*** *source of metadata (links or references):*  Websites ref.Nr.^115,116^ link to the following publication: ^111^ | *As* ***direct*** *source of metadata:*  Blood pressure, bone ultrasonography, blood samples (no details provided) ^115^;  *As* ***indirect*** *source of metadata (links or references):*  Websites ref.Nr.^115,116^ link to the following publication: ^111,112^ | *As* ***indirect*** *source of metadata (links or references):*  Websites ref.Nr.^115,116^ link to the following publication: ^111^ |
|  | Study/trial registries | *As* ***direct*** *source of metadata:*  Nutritional, metabolic, endocrine (no distinction prevalent/incident) ^33^; prevalence of overweight and obesity ^117^;  *As* ***indirect*** *source of metadata (links or references):*  Registries ref.Nr ^35,117,118^ link to website and the following publications: ^110-112^ | *As* ***indirect*** *source of metadata (links or references):*  NFDI4Health COVID-19 study portal ^117^ links to the following publication: ^111^ | *As* ***direct*** *source of metadata:*  Primary: overweight/obesity, metabolic syndrome, bone stiffness/bone health (full list of primary and secondary outcomes) ^33,118^; obesity, overweight, metabolic disorders ^35^;  *As* ***indirect*** *source of metadata (links or references):*  Registries ref.Nr ^35,117,118^ link to website and the following publications: ^110,111^ | *As* ***indirect*** *source of metadata (links or references):*  Registries ref.Nr ^117,118^ link to website and the following publications: ^110-112^ | *As* ***indirect*** *source of metadata (links or references):*  Registries ^35,117^ link to website and/or the following publication: ^111^ | *As* ***indirect*** *source of metadata (links or references):*  NFDI4Health COVID-19 study portal ^117^ links to the following publication: ^111^ | *As* ***indirect*** *source of metadata (links or references):*  NFDI4Health COVID-19 study portal ^117^ links to the following publication: ^111^ | *As* ***direct*** *source of metadata:*  Fasting blood, urine samples, blood pressure (no details provided) ^118^  *As* ***indirect*** *source of metadata (links or references):*  Registries ref.Nr ^35,117,118^ link to website and the following publications: ^110-112^ | *As* ***direct*** *source of metadata:*  Medical records ^117^;  *As* ***indirect*** *source of metadata (links or references):*  Registries ref.Nr ^35,117,118^ link to website and the following publications: ^110,111^ |
|  | Data documents | Health and medical history interview form (T3: third follow-up) includes list of diseases to ask about parents, index child, and siblings ^111^ | - | - | Health and medical history interview form (T3: third follow-up): CAPI/CATI ^111^ | Health and medical history interview form (T3: third follow-up): Diagnosis, age at diagnosis, hospitalization, therapy, medication ^111^ | Health and medical history interview form (T3: third follow-up): Ever (diagnosis), last 14 days (medication) ^111^ | - | Examinations recording sheet (T3): blood pressure and pulse rate ^111^;  Laboratory documentation sheet (SOP, T3): blood samples ^111^; | - |
| KORA | Scientific publications | Prevalent diseases (incl. CVDs and T2D)^119^;  Incident T2D ^120,121^;  Prevalent and incident CVDs (fatal and nonfatal), all-cause mortality ^122^ | ICD-10 from death certificates ^119,122^;  Incident colorectal cancer coded with ICD-9 or ICD-10 ^123^ | Primary: acute coronary events (AMI attacks and CHD deaths) ^124^; multimorbidity ^119^ | Paper-based self-completed questionnaire at baseline (full sample) ^119^;  CAPI (at baseline and FU of KORA-Age subsample) ^123,125^ | Diagnosis and medications: CVDs, T2D, cancer (paper-based, self-completed) ^119^;  Diabetes: diagnosis, medication ^120,121,126^;  Hypertension: based on study examinations (blood pressure) or antihypertensive medication ^126^ | Ever (CVDs), last 3 years (cancer) ^127^;  Last 7 days (medication) ^119,126^ | Prevalent and incident T2D verified by medical records/general practitioner ^120,121^;  Incident cancer verified with cancer registry data ^123^ | Blood pressure (cut-off for hypertension included) ^121,122,126,128^, measurement protocol ^121^;  Diabetes and prediabetes based on OGTT and fasting glucose ^121,128^;  Blood samples (laboratory measurements included HbA1c, total cholesterol, HDL-C, LDL-C) ^126^  Cardiovascular magnetic resonance (CMR) to examine subclinical CVD alterations ^126^;  MRI to examine carotid plaque and cardiac function ^128^ | Death certificates ^119,122,124,125^;  Myocardial Infarction Registry in Augsburg ^124,125^ |
|  | Study website | *As* ***direct*** *source of metadata:*  CVDs, chronic diseases ^129^;  *As* ***indirect*** *source of metadata (links or references):*  Website ref.Nr ^129^ links to the following publications: ^122^ ^119,120^ | *As* ***indirect*** *source of metadata (links or references):*  Website ref.Nr ^129^ links to the following publications: ^119,122,123^ | *As* ***direct*** *source of metadata:*  Primary: Mainly CVDs; some FU surveys have also focused on diabetes and cancer ^129^;  *As* ***indirect*** *source of metadata (links or references):*  Website ref.Nr ^129^ links to the following publications: ^119,124^ | *As* ***direct*** *source of metadata:*  KORA FFF4: self-completed, both paper- and computer-based are available ^129^;  *As* ***indirect*** *source of metadata (links or references):*  Website ref.Nr ^129^ links to the following publications: ^119,123,125^ | *As* ***indirect*** *source of metadata (links or references):*  Website ref.Nr ^129^ links to the following publications: ^119-121,126^ | *As* ***indirect*** *source of metadata (links or references):*  Website ref.Nr ^129^ links to the following publications: ^126,127^ | *As* ***indirect*** *source of metadata (links or references):*  Website ref.Nr ^129^ links to the following publications: ^120,123^ | *As* ***direct*** *source of metadata:*  KORA FFF4: blood pressure, OGTT, blood samples (no details provided) ^129^;  *As* ***indirect*** *source of metadata (links or references):*  Website ref.Nr ^129^ links to the following publications: ^121,122,126,128^ | *As* ***indirect*** *source of metadata (links or references):*  Website ref.Nr ^129^ links to the following publications: ^119,122,124,125^ |
|  | Study/trial registries | *As* ***direct*** *source of metadata:*  Prevalent (at baseline): MI, angina pectoris, stroke, diabetes, heart failure, cancer ^130^; Incident (followed up end-points): myocardial infarction, stroke, diabetes ^93,130^;  CVD-related diseases (list)^93^;  *As* ***indirect*** *source of metadata (links or references):*  Registries ref.Nr ^85,93,94,131^ link to study website ^129^ and/or the following publication: ^122^ | *As* ***direct*** *source of metadata:*  Validated outcomes and fatal outcomes with ICD-9 ^130^;  *As* ***indirect*** *source of metadata (links or references):*  Registries ref.Nr ^85,93,94,131^ link to study website ^129^ and/or the following publication: ^122^ | *As* ***direct*** *source of metadata:*  Primary: myocardial infarction and T2D ^93^;  *As* ***indirect*** *source of metadata (links or references):*  Registries ref.Nr ^85,93,94,131^ link to study website ^129^ and/or the following publication: ^124^ | *As* ***direct*** *source of metadata:*  Face-to-face interview (baseline) self-completed paper-based (passive FU) ^94^;  *As* ***indirect*** *source of metadata (links or references):*  Registries ref.Nr ^85,93,94,130,131^ link to study website ^129^ and/or the following publication: ^125^ | *As* ***direct*** *source of metadata:*  Diagnosis/disease, treatment ^130^; medical history, medication ^94^;  *As* ***indirect*** *source of metadata (links or references):*  Registries ref.Nr ^85,93,94,131^ link to study website ^129^ | *As* ***direct*** *source of metadata:*  Ever (prevalent MI, angina pectoris, stroke, diabetes, cancer), last 12 months (prevalent heart failure, cancer) ^130^ | *As* ***direct*** *source of metadata:*  Clinical outcomes reported in passive FU validated by contacting physicians and/or chart review ^94^; self-reported incident stroke and diabetes validated by hospital records or contacting treating physician ^130^; | *As* ***direct*** *source of metadata:*  Rose questionnaire ^130^; blood (baseline and selected FU examinations) ^93,94^; list of biological samples available and storage conditions ^131^;  *As* ***indirect*** *source of metadata (links or references):*  Registries ref.Nr ^85,93,94,131^ link to study website ^129^ and/or the following publication: ^122^ | *As* ***direct*** *source of metadata:*  Vital statistics from population registries ^93,94^; population registries, regional health departments, coronary events registry ^130^; medical records, national registries ^131^;  *As* ***indirect*** *source of metadata (links or references):*  Registries ref.Nr ^85,93,94,130,131^ link to study website ^129^ and/or the following publications: ^122,124,125^ |
|  | Data documents | Available from variable lists and tables ^132^ | ICD-9 and ICD-10 available for cancer ^132^ | - | Variables indicating whether surveys were self-completed or telephone ^132^ | Variables with questions: diagnosis, treatment/medication, complications ^132^ | Variables with questions: ever (hypertension, MI, stroke, arrhythmias, angina pectoris, circulatory disorders, CAD, PAD, cancer); in the last 12 months (heart failure, cancer); last 7 days (medications) ^132^ | Variables for verified diabetes ^132^ | Variables for blood pressure and heart rate (average of second and third measures), laboratory measurements and imagery data ^132^;  Definitions for study examination-based disease ascertainment: hypertension, angina pectoris, pre-diabetes, diabetes ^132^ | - |
| lidA | Scientific publications | - | ICD coded medical diagnoses from health insurance data ^133^ | Primary: subjective health status, physical health, mental health, depression, grip strength ^133^ | CAPI ^133^ | - | - | Data linkage used to check validity (cross-validating self-reported health in the study) ^133^ | na. | Linked with health insurance claims data ^133^ |
|  | Study website | - | *As* ***indirect*** *source of metadata (links or references):*  Website ref.Nr ^134^ links to the following publication: ^133^ | *As* ***indirect*** *source of metadata (links or references):*  Website ref.Nr ^134^ links to the following publication: ^133^ | *As* ***direct*** *source of metadata:*  Face-to-face interview (home visits), telephone interview (wave 4, 2022) ^134^;  *As* ***indirect*** *source of metadata (links or references):*  Website ref.Nr links to the following publication: ^133^ | - | - | *As* ***indirect*** *source of metadata (links or references):*  Website ref.Nr ^134^ links to the following publication: ^133^ | na. | *As* ***direct*** *source of metadata:*  Linkage to federal employment agency (all waves), health insurance (waves 1,2), pension insurance (waves 3, 4) ^134^;  *As* ***indirect*** *source of metadata (links or references):*  Website ref.Nr ^134^ links to the following publication: ^133^ |
|  | Registries | na. | na. | na. | na. | na. | na. | na. | na. | na. |
|  | Data documents | Questions indicate both prevalent and incident outcomes are available ^135^ | - | - | CAPI ^135^ | Diagnosis ^135^ | CVDs, internal diseases, others: Ever (wave 1), since last interview (wave 2), last 12 months (waves 1 and 2) ^135^ | - | na. | - |
| LIFE-Adult | Scientific publications | List of prevalent outcomes ^136^;  Prevalence and incidence of common diseases, including CVDs and metabolic disorders (detailed list not provided) ^137^ | - | Primary: CVDs (MI, HF, atherosclerosis, cardiac arrhythmias), metabolic disorders (obesity, T2D), others ^136^; prevalence and incidence of common diseases, focus on some disease groups incl. CVDs and metabolic disorders ^137^ | Prevalent (baseline): CAPI ^136,137^;  Incident (passive follow-up): self-completed paper- or computer-based ^137^;  Incident (active follow-up, subsample): medical history self-completed paper/computer-based and  medications by CAPI ^137^ | At baseline (prevalent outcomes): Diagnosis, symptoms, treatment ^136^;  Prevalent and incident (baseline and follow-up): medical history, medications ^137^ | At baseline (prevalent outcomes): ever (diagnosis), last 12 months (symptoms), current (treatment) ^136^;  Prevalent and incident (baseline and follow-up): last 7 days (medication) ^137^ | Self-reported changes of health status validated by confirmation from treating physician ^137^;  Diabetes: plan to supplement study data with records from health insurance (verification planned in the future) ^138^ | All examinations at baseline (prevalent outcomes) explained in detail ^136^;  Examinations at baseline: blood samples, blood pressure, ECG, echocardiography, carotid ultrasound, OGTT (subsample) ^137^;  Examinations in a subsample at follow-up: blood samples, ECG, carotid ultrasound, ABI, 7-day ECG ^137^;  Laboratory analyses included cardiac markers, markers of lipid and glucose metabolism ^137^ | Health insurance data linkage, vital status updated from public population register ^137^ |
|  | Study website | *As* ***indirect*** *source of metadata (links or references):*  Website ref.Nr ^139^ lists the following publication: ^136^ | - | *As* ***direct*** *source of metadata:*  CVDs among primary endpoints ^139^;  *As* ***indirect*** *source of metadata (links or references):*  Website ref.Nr ^139^ lists the following publication: ^136^ | *As* ***direct*** *source of metadata:*  Incident (passive FU): Self-completed paper-based; Incident (re-examination): same as baseline ^139^;  *As* ***indirect*** *source of metadata (links or references):*  Website ref.Nr ^139^ lists the following publication: ^136^ | *As* ***indirect*** *source of metadata (links or references):*  Website ref.Nr ^139^ lists the following publication: ^136^ | *As* ***indirect*** *source of metadata (links or references):*  Website ref.Nr ^139^ lists the following publication: ^136^ | *As* ***indirect*** *source of metadata (links or references):*  Website ref.Nr ^139^ lists the following publication: ^138^ | *As* ***direct*** *source of metadata:*  Blood sample (baseline and follow-up), abdominal MRT (subsample) ^139^;  Incident diseases: all examinations at re-examination are the same as baseline ^139^;  *As* ***indirect*** *source of metadata (links or references):*  Website ref.Nr ^139^ lists the following publication: ^136^ | - |
|  | Study/trial registries | *As* ***indirect*** *source of metadata (links or references):*  Leipzig Health Atlas ^140^ links to website ^139^ | - | *As* ***direct*** *source of metadata:*  Common diseases such as cancer, diabetes, CVDs ^140^;  *As* ***indirect*** *source of metadata (links or references):*  Leipzig Health Atlas ^140^ links to website ^139^ | *As* ***indirect*** *source of metadata (links or references):*  Leipzig Health Atlas ^140^ links to website ^139^ | *As* ***indirect*** *source of metadata (links or references):*  Leipzig Health Atlas ^140^ links to website ^139^ | *As* ***indirect*** *source of metadata (links or references):*  Leipzig Health Atlas ^140^ links to website ^139^ | *As* ***indirect*** *source of metadata (links or references):*  Leipzig Health Atlas ^140^ links to website ^139^ | *As* ***indirect*** *source of metadata (links or references):*  Leipzig Health Atlas ^140^ links to website ^139^ | - |
|  | Data documents | Baseline annotated case report forms (data dictionaries): hypertension, vascular diseases, MI, CHD, stroke, arterial disease, T2D, arrhythmias, HF, cancer ^141^;  Follow-up case report forms (incident data) not yet available but items visible. Same diseases as prevalent asked ^141^ | - | - | Face-to-face interview (baseline)^141^;  Self-completed (passive and active follow-up) ^141^ | Questions available on case report forms:  T2D: disease, medications; CVDs: disease, symptoms; cancer: disease, treatment, resolution ^141^ | Questions available on case report forms: ever (disease, symptoms), last 12 months (disease), 4 weeks (symptoms), current (symptoms) ^141^ | - | Baseline annotated case report forms (data dictionaries) specific to various study examinations: carotid sonography for Intima Media thickness; ABI; blood pressure; diabetes status according to: HbA1c, fasting glucose, OGTT; atrial fibrillation and flutter, arrhythmias based on ECG evaluation ^141^ | - |
| NAKO | Scientific publications | Prevalent and incident: MI, HF, stroke, atrial fibrillation, PAD, diabetes mellitus, cancer ^142,143^ | Yes, only incident (and lethal) outcomes ^142^ | Chronic diseases (CVDs, diabetes mellitus, cancer, others) ^142,143^ | Prevalent and incident (at re-examination): CAPI ^142-144^;  Incident (active follow-up): self-completed paper-based ^143,145^ | Prevalent and incident: diagnosis, treatment ^145^ | Ever (diagnosis), last year (treatment) ^144,145^ | Incident: contacting treating physician and/or hospitals where treated ^142^ | Prevalent and incident (at re-examination) CVDs: arterial stiffness, ankle-brachial index, intima-media thickness of carotid artery, MRI, ECG, 3D-echocardiography, MRI, blood pressure ^142,143^;  Prevalent and incident (at re-examination): diabetes: fasting glucose, OGTT, skin autofluorescence, retinal photographs ^142,143^;  Prevalent and incident (at re-examination) cancer: precursor stages of haematologic malignancies ^142^ | Incident cancer: record linkage with cancer registries ^142,143,145^;  Incident non-cancer outcomes: linkage to health insurance records (other diseases) ^142,143^ |
|  | Study website | *As* ***indirect*** *source of metadata (links or references):*  Website ref.Nr ^146^ lists the following publications: ^142,143^ | *As* ***indirect*** *source of metadata (links or references):*  Website ref.Nr ^146^ lists the following publication: ^142^ | *As* ***direct*** *source of metadata:*  Primary: chronic diseases including cancer, diabetes, CVDs ^146^;  *As* ***indirect*** *source of metadata (links or references):*  Website ref.Nr ^146^ lists the following publications: ^142,143^ | *As* ***direct*** *source of metadata:*  Incident (follow-up): self-completed, paper-based ^146^;  *As* ***indirect*** *source of metadata (links or references):*  Website ref.Nr ^146^ lists the following publications: ^142-145^ | *As* ***indirect*** *source of metadata (links or references):*  Website ref.Nr ^146^ lists the following publication: ^145^ | *As* ***indirect*** *source of metadata (links or references):*  Website ref.Nr ^146^ lists the following publications: ^144,145^ | *As* ***direct*** *source of metadata:*  Incident: contacting treating physician ^146^;  *As* ***indirect*** *source of metadata (links or references):*  Website ref.Nr ^146^ lists the following publication: ^142^ | *As* ***direct*** *source of metadata:*  Blood pressure, heart rate, MRT, blood samples (details not provided) ^146^;  *As* ***indirect*** *source of metadata (links or references):*  Website ref.Nr ^146^ lists the following publications: ^142,143^ | *As* ***indirect*** *source of metadata (links or references):*  Website ref.Nr ^146^ lists the following publications: ^142,143,145^ |
|  | Study/trial registries | *As* ***direct*** *source of metadata:*  List of diseases, including CDVs, T2D, cancer (no specification of prevalence/incidence) ^36^;  *As* ***indirect*** *source of metadata (links or references):*  Registries ^36,85,147^ link to study website ^146^ and/or the following publication: ^142^ | *As* ***indirect*** *source of metadata (links or references):*  Registries ^36,85,147^ link to study website ^146^ and/or the following publication: ^142^ | *As* ***direct*** *source of metadata:*  Primary: chronic diseases including cancer, diabetes, CVDs ^85,147^;    *As* ***indirect*** *source of metadata (links or references):*  Registries ^36,85,147^ link to study website ^146^ and/or the following publication: ^142^ | *As* ***direct*** *source of metadata:*  CAPI ^36^;  *As* ***indirect*** *source of metadata (links or references):*  Registries ^36,85,147^ link to study website ^146^ and/or the following publication: ^142^ | *As* ***indirect*** *source of metadata (links or references):*  Registries ^36,85,147^ link to study website ^146^ | *As* ***indirect*** *source of metadata (links or references):*  Registries ^36,85,147^ link to study website ^146^ | *As* ***indirect*** *source of metadata (links or references):*  Registries ^36,85,147^ link to study website ^146^ and/or the following publication: ^142^ | *As* ***direct*** *source of metadata:*  Blood samples, HDL-C, LDL-C, total cholesterol, triglycerides, glucose, HbA1c (no details provided) ^36^;  *As* ***indirect*** *source of metadata (links or references):*  Registries ^36,85,147^ link to study website ^146^ and/or the following publication: ^142^ | *As* ***direct*** *source of metadata:*  Health and retirement insurances and mortality registries ^36^;  *As* ***indirect*** *source of metadata (links or references):*  Registries ^36,85,147^ link to study website ^146^ and/or the following publication: ^142^ |
|  | Data documents | Available from data dictionary ^148^ | ICD code is a variable for cause of death (data dictionary) ^148^ | - | Data dictionary ^148^ specifies which variables are from interview (paper/computer-based not specified) and which from paper-based questionnaire) | Data dictionary ^148^ includes questions (diagnosis, treatment) | Data dictionary ^148^ includes questions (ever diagnosed, treated in last 12 mo) | - | Data dictionary ^148^ includes variables for medical examination measures and time points (baseline or follow-up) | - |
| SHIP/SHIP Trend | Scientific publications | Both prevalence and incidence of chronic diseases ^149,150^; list of diseases and examinations at which they were assessed ^150^ | From linked health insurance data ^151,152^; from stationary treatments of the Greifswald University Medical Center ^152^; from death certificates ^153^ | Wide focus ^150,152,154^ | Prevalent and incident (at re-examination): CAPI ^149,151,154-156^;  Incident (passive follow-up): self-completed paper-based or CATI ^157^ | Diagnosis, medication ^151,158,159^, cardiovascular symptoms ^160^ | Last 7 days (medication) ^159^ | Only incident outcomes are validated by GPs ^152,154^ | List and description of all examinations ^150,154^;  Diabetes: glucose concentration cut-off ^161^ and definitions (T2D, prediabetes) based on OGTT, HbA1c and fasting glucose ^162^;  Blood pressure (description, cut-offs for hypertension), HbA1c (cut-offs for diabetes) ^158,163^;  Blood pressure, ECG, OGTT, carotid artery ultrasound, echocardiography, ABI (no details) ^149^;  Carotid atherosclerotic plaques defined based on results of ultrasonography ^163^;  Left ventricular hypertrophy defined based on electrocardiographic examination, hypercholesterolemia defined based on total serum cholesterol and/or LDL-C and/or total cholesterol/HDL-C ratio ^164^;  Aortic stenosis defined based on echocardiography examination ^165^ | Linked to health insurance data ^150,151^, population registries, health agencies hospitals ^150^;  Database of the Greifswald University Medical Centre (e.g., biopsy reports) ^166^;  Death certificates obtained once a year ^158^;  Description of all sources of secondary data ^152^ |
|  | Study website | *As* ***direct*** *source of metadata:*  Prevalence and incidence of chronic diseases (incl. CVDs, diabetes mellitus, cancer) ^167^;  *As* ***indirect*** *source of metadata (links or references):*  Website ref.Nr ^167^ links to the following publications: ^149,161^, and to data documents (baseline interview form) | - | *As* ***direct*** *source of metadata:*  Primary: CVDs, diabetes mellitus, liver and gallbladder diseases, neurological diseases, thyroid disease, dental diseases, lung disease ^167^;  *As* ***indirect*** *source of metadata (links or references):*  Website ref.Nr ^167^ links to the following publication: ^154^ | *As* ***indirect*** *source of metadata (links or references):*  Website ref.Nr ^167^ links to data documents (baseline interview form) | *As* ***indirect*** *source of metadata (links or references):*  Website ref.Nr ^167^ links to data documents (baseline interview form) | *As* ***indirect*** *source of metadata (links or references):*  Website ref.Nr ^167^ links to data documents (baseline interview form) | *As* ***indirect*** *source of metadata (links or references):*  Website ref.Nr ^167^ links to the following publication: ^154^ | *As* ***indirect*** *source of metadata (links or references):*  Website ref.Nr ^167^ links to the following publications: ^149,154,161^, and to data documents (baseline data entry forms for all examinations) ^167^ | - |
|  | Study/trial registries | *As* ***direct*** *source of metadata:*  Both prevalence and incidence of chronic diseases (list of diseases) ^93,94^;  *As* ***indirect*** *source of metadata (links or references):*  Registries ^35,85,93,94,147^ link to website ^167^, and the following publication: ^149,150,154^ | *As* ***direct*** *source of metadata:*  ICD-10 codes for cause of death ^130^ | *As* ***direct*** *source of metadata:*  Primary: chronic diseases (broad aim) ^35,93,94^;  *As* ***indirect*** *source of metadata (links or references):*  Registries ^35,85,93,94,147^ link to website ^167^ and the following publications: ^150,154^ | *As* ***direct*** *source of metadata:*  Incident (passive FU): self-completed paper-based ^93,94^;  *As* ***indirect*** *source of metadata (links or references):*  Registries ^35,85,93,94,147^ link to website ^167^ and the following publications: ^149,154^ | *As* ***direct*** *source of metadata:*  Includes variable list with domain in label ^94^; MDM Portal includes interview questions^12^;  *As* ***indirect*** *source of metadata (links or references):*  Registries ^35,85,93,94,147^ link to website ^167^ | *As* ***direct*** *source of metadata:*  Includes variable list with reference period in label ^94^; MDM Portal includes interview questions^12^;  *As* ***indirect*** *source of metadata (links or references):*  Registries ^35,85,93,94,147^ link to website ^167^ | *As* ***direct*** *source of metadata:*  Incident (written FU): validated by GP survey ^93,94^;  *As* ***indirect*** *source of metadata (links or references):*  Registries ^35,94^ link to the following publication: ^154^ | *As* ***direct*** *source of metadata:*  Variables of measured diseases/disease markers ^94^;  *As* ***indirect*** *source of metadata (links or references):*  Registries ^35,85,93,94,147^ link to website ^167^ and the following publication: ^149,150,154^ | *As* ***direct*** *source of metadata:*  Outpatient treatment data obtained from panel physicians association. Clinical data from Greifswald University Medical Centre and health insurances ^93,94^;  *As* ***indirect*** *source of metadata (links or references):*  Registries ^35,93^ link to the following publication: ^150^ |
|  | Data documents | Prevalent and incident: list of diseases available through baseline interview form ^167^ and data dictionaries (baseline and follow-up) ^168^ | Data dictionary with ICD-10 for cause of death ^168^ | - | CAPI (medical history questions found in baseline interview form) ^167^ | Diagnosis/disease, symptoms, treatment/medications ^167,168^ | Questions available in baseline interview form  ^167^ and data dictionaries ^168^; | - | Prevalent (at baseline) and incident (at re-examination) CVDs: ECG, echocardiogram, carotid sonography, blood pressure;  Prevalent and incident (at re-examination) T2D: blood glucose, HBA1c ^167^ | - |

ABI, Ankle Brachial Index; AMI, Acute Myocardial Infarction; CAD, Coronary Artery Disease; CAPI, Computer Assisted Personal Interview; CATI, Computer Assisted Telephone Interview; CHD, Coronary Heart Disease; CRP, C-Reactive Protein; CVD, Cardiovascular Disease; EBCT, Electro Beam Computed Tomography; ECG, Electrocardiogram; FU, Follow-up; HDL-C, High Density Lipoprotein Cholesterol; HF, Heart Failure; LDL-C, Low Density Lipoprotein Cholesterol; MI, Myocardial Infarction; MRI, Magnetic Resonance Imaging; OGTT, Oral Glucose Tolerance Test; PAD, Peripheral Artery Disease; T2D, Type 2 Diabetes Mellitus.

-, missing metadata; na., not applicable (i.e., source not found or metadata field not applicable to study design).

^a^ Not considered if consulted for case verification only; considered if may be consulted for or complemented disease ascertainment (e.g., cause of death from death certificates to complement disease incidence data).

# Supplementary Table 3: The FAIR Guiding Principles, applied to metadata^a^

| Criteria for Findability: |
| --- |
| F1. Metadata are assigned a globally unique and persistent identifier |
| F2. Rich metadata describe the data |
| F3. Metadata clearly and explicitly include the identifier of the data it describes |
| F4. Metadata are registered or indexed in a searchable resource |
| Criteria for Accessibility: |
| A1. Metadata are retrievable by their identifier using a standardised communications protocol |
| A1.1. The protocol is open, free, and universally implementable |
| A1.2. The protocol allows for an authentication and authorisation procedure, where necessary |
| A2. Metadata are accessible, even when the data are no longer available |
| Criteria for Interoperability: |
| I1. Metadata use a formal, accessible, shared, and broadly applicable language for knowledge representation (standard ontology) |
| I2. Metadata use vocabularies that follow FAIR principles (standard terminology) |
| I3. Metadata include qualified references to other metadata (hierarchical structure of metadata) |
| Criteria for Reusability: |
| R1. Metadata are richly described with a plurality of accurate and relevant attributes |
| R1.1. Metadata are released with a clear and accessible data usage license |
| R1.2. Metadata are associated with detailed provenance |
| R1.3. Metadata meet domain-relevant community standards |

^a^ Adapted from Wilkinson, et al. (2016). <https://doi.org/10.1038/sdata.2016.18>^169^

# Supplementary Table 4: Other perceived barriers to achieve FAIR (meta-)data

| **Barrier** | **Very important barrier** | **Moderately important barrier** | **Not an important barrier** |
| --- | --- | --- | --- |
| High complexity of study design (staff need a lot of historic knowledge and up to date data management skills) | X |  |  |
| Standards, best practice tools missing. Project-specific harmonisation use up resources but are often not re-usable | X |  |  |
| Documentation is sometimes hard to find (e.g., laboratory parameters or for aggregated data, especially if several people worked on it or left before finishing) |  | X |  |
| Person-related data require extra protection and cannot be anonymised effectively | X |  |  |
| Multi-national multi-centric data requires confirmation of each release by each data provider | X |  |  |
| Limited data quality | X |  |  |

# Supplementary references

1 Greiser, K. H. *et al.* Cardiovascular disease, risk factors and heart rate variability in the elderly general population: Design and objectives of the CARdiovascular disease, Living and Ageing in Halle (CARLA) Study. *BMC Cardiovascular Disorders* **5**, 33 <https://doi.org/10.1186/1471-2261-5-33> (2005).

2 Greiser, K. H. *et al.* Cardiovascular diseases, risk factors and short-term heart rate variability in an elderly general population: the CARLA study 2002–2006. *European Journal of Epidemiology* **24**, 123 <https://doi.org/10.1007/s10654-009-9317-z> (2009).

3 Hassan, L. *et al.* Cardiovascular risk factors, living and ageing in Halle: the CARLA study. *European Journal of Epidemiology* **37**, 103-116 <https://doi.org/10.1007/s10654-021-00824-7> (2022).

4 Schott, A. *et al.* Association of arterial stiffness and heart failure with preserved ejection fraction in the elderly population – results from the CARLA study. *Journal of Human Hypertension* <https://doi.org/10.1038/s41371-022-00703-y> (2022).

5 Herrmann, W. J. *et al.* Erfassung inzidenter kardiovaskulärer und metabolischer Erkrankungen in epidemiologischen Kohortenstudien in Deutschland [Recording of incident cardiovascular and metabolic diseases in epidemiological cohort studies in Germany]. *Bundesgesundheitsblatt - Gesundheitsforschung - Gesundheitsschutz* **61**, 420-431 <https://doi.org/10.1007/s00103-018-2712-4> (2018).

6 Hassan, L. *et al.* The association between change of soluble tumor necrosis factor receptor R1 (sTNF-R1) measurements and cardiovascular and all-cause mortality—Results from the population-based (Cardiovascular Disease, Living and Ageing in Halle) CARLA study 2002–2016. *PLOS ONE* **15**, e0241213 <https://doi.org/10.1371/journal.pone.0241213> (2020).

7 Lacruz, M. E. *et al.* Prevalence and Incidence of Hypertension in the General Adult Population: Results of the CARLA-Cohort Study. *Medicine (Baltimore)* **94**, e952-e952 <https://doi.org/10.1097/MD.0000000000000952> (2015).

8 Univeristy Hospital Halle (Saale). *Kardiovaskuläre Risikofaktoren und Krankheiten in der älteren Allgemeinbevölkerung - Die CARLA-Studie [Cardiovascular risk factors and diseases in the elderly general population - The CARLA study]*, <https://www.medizin.uni-halle.de/en/einrichtungen/institute/medizinische-epidemiologie-biometrie-und-informatik/forschung/ag-chronische-erkrankungen/projekte/carla-studie/carla>

9 Univeristy Hospital Halle (Saale) & University Medicine Martin-Luther-University Halle-Wittenberg. *Forms and Instruments [CARLA]*, <https://webszh.uk-halle.de/carla-studie/index.php/formulare-und-instrumente/>

10 Univeristy Hospital Halle (Saale) & University Medicine Martin-Luther-University Halle-Wittenberg. *Welcome to the CARLA-Study: "Healthy living with heart" in Halle, Germany*, <https://webszh.uk-halle.de/carla-studie/>

11 Univeristy Hospital Halle (Saale) & University Medicine Martin-Luther-University Halle-Wittenberg. *Data Dictionaries [CARLA]*, <https://webszh.uk-halle.de/carla-studie/index.php/variablenverzeichnisse/>

12 *Medical Data Models: MDM Portal*, <https://medical-data-models.org/> (2023).

13 Scheidt-Nave, C. *et al.* German health interview and examination survey for adults (DEGS) - design, objectives and implementation of the first data collection wave. *BMC Public Health* **12**, 730 <https://doi.org/10.1186/1471-2458-12-730> (2012).

14 Kamtsiuris, P. *et al.* Die erste Welle der Studie zur Gesundheit Erwachsener in Deutschland (DEGS1) [The first wave of the German Health Interview and Examination Survey for Adults (DEGS1)]. *Bundesgesundheitsblatt - Gesundheitsforschung - Gesundheitsschutz* **56**, 620-630 <https://doi.org/10.1007/s00103-012-1650-9> (2013).

15 Gößwald, A., Lange, M., Kamtsiuris, P. & Kurth, B. M. DEGS: Studie zur Gesundheit Erwachsener in Deutschland [DEGS: German Health Interview and Examination Survey for Adults]. *Bundesgesundheitsblatt - Gesundheitsforschung - Gesundheitsschutz* **55**, 775-780 <https://doi.org/10.1007/s00103-012-1498-z> (2012).

16 Busch, M. A., Schienkiewitz, A., Nowossadeck, E. & Gößwald, A. Prävalenz des Schlaganfalls bei Erwachsenen im Alter von 40 bis 79 Jahren in Deutschland [Prevalence of stroke in adults aged 40 to 79 in Germany]. *Bundesgesundheitsblatt - Gesundheitsforschung - Gesundheitsschutz* **56**, 656-660 <https://doi.org/10.1007/s00103-012-1659-0> (2013).

17 Heidemann, C., Du, Y., Schubert, I., Rathmann, W. & Scheidt-Nave, C. Prävalenz und zeitliche Entwicklung des bekannten Diabetes mellitus [Prevalence and development over time of known diabetes mellitus]. *Bundesgesundheitsblatt - Gesundheitsforschung - Gesundheitsschutz* **56**, 668-677 <https://doi.org/10.1007/s00103-012-1662-5> (2013).

18 Gößwald, A., Schienkiewitz, A., Nowossadeck, E. & Busch, M. A. Prävalenz von Herzinfarkt und koronarer Herzkrankheit bei Erwachsenen im Alter von 40 bis 79 Jahren in Deutschland [Prevalence of myocardial infarction and coronary artery disease in adults aged 40 to 79 years in Germany]. *Bundesgesundheitsblatt - Gesundheitsforschung - Gesundheitsschutz* **56**, 650-655 <https://doi.org/10.1007/s00103-013-1666-9> (2013).

19 *DEGS. Studie zur Gesundheit Erwachsener in Deutschland: Eine Studie des Robert-Koch-Instituts [DEGS. German Health Interview and Examination Survey for Adults: A Study of the Robert Koch Institute]*, <https://www.degs-studie.de/deutsch/home.html>

20 Robert Koch Institute. *DEGS: Studie zur Gesundheit Erwachsener in Deutschland [DEGS: German Health Interview and Examination Survey for Adults]*, <https://www.rki.de/DE/Content/Gesundheitsmonitoring/Studien/Degs/degs_node.html>

21 Robert Koch Institute. *Datenangebot des Forschungsdatenzentrums [Data offer of the Research Data Center]*, <https://www.rki.de/DE/Content/Forsch/FDZ/Datenangebot/Datenangebot_node.html;jsessionid=8534447A31D0DAFAFCD994CA8EBE4D3E.internet112>

22 Buyken, A. E., Alexy, U., Kersting, M. & Remer, T. Die DONALD Kohorte [The DONALD cohort]. *Bundesgesundheitsblatt-Gesundheitsforschung-Gesundheitsschutz* **55**, 875-884 <https://doi.org/10.1007/s00103-012-1503-6> (2012).

23 Della Corte, K. A. *et al.* The Prospective Association of Dietary Sugar Intake in Adolescence With Risk Markers of Type 2 Diabetes in Young Adulthood. *Frontiers in Nutrition* **7** <https://doi.org/10.3389/fnut.2020.615684> (2021).

24 Goletzke, J. *et al.* Habitually Higher Dietary Glycemic Index During Puberty Is Prospectively Related to Increased Risk Markers of Type 2 Diabetes in Younger Adulthood. *Diabetes Care* **36**, 1870-1876 <https://doi.org/10.2337/dc12-2063> (2013).

25 Krupp, D., Shi, L. & Remer, T. Longitudinal relationships between diet-dependent renal acid load and blood pressure development in healthy children. *Kidney International* **85**, 204-210 <https://doi.org/10.1038/ki.2013.331> (2014).

26 Krupp, D., Westhoff, T. H., Esche, J. & Remer, T. Prospective relation of adolescent citrate excretion and net acid excretion capacity with blood pressure in young adulthood. *American Journal of Physiology-Renal Physiology* **315**, F1228-F1235 <https://doi.org/10.1152/ajprenal.00144.2018> (2018).

27 Nyasordzi, J., Penczynski, K., Remer, T. & Buyken, A. E. Early life factors and their relevance to intima-media thickness of the common carotid artery in early adulthood. *PLOS ONE* **15**, e0233227 <https://doi.org/10.1371/journal.pone.0233227> (2020).

28 Penczynski, K. J. *et al.* Flavonoid intake from fruit and vegetables during adolescence is prospectively associated with a favourable risk factor profile for type 2 diabetes in early adulthood. *European Journal of Nutrition* **58**, 1159-1172 <https://doi.org/10.1007/s00394-018-1631-3> (2019).

29 Shi, L., Krupp, D. & Remer, T. Salt, fruit and vegetable consumption and blood pressure development: a longitudinal investigation in healthy children. *British Journal of Nutrition* **111**, 662-671 <https://doi.org/10.1017/S0007114513002961> (2014).

30 Oluwagbemigun, K. *et al.* Developmental trajectories of body mass index from childhood into late adolescence and subsequent late adolescence–young adulthood cardiometabolic risk markers. *Cardiovascular Diabetology* **18**, 9 <https://doi.org/10.1186/s12933-019-0813-5> (2019).

31 Schnermann, M. E., Schulz, C.-A., Herder, C., Alexy, U. & Nöthlings, U. A lifestyle pattern during adolescence is associated with cardiovascular risk markers in young adults: results from the DONALD cohort study. *Journal of Nutritional Science* **10**, e92 <https://doi.org/10.1017/jns.2021.84> (2021).

32 *DONALD Studie: Studiendesign und Methoden [The Donald study: study design and methods]*, <https://www.ernaehrungsepidemiologie.uni-bonn.de/forschung/donald-1/studiendesign>

33 *International Clinical Trials Registry Platform (ICTRP) Search Portal*, <https://trialsearch.who.int/> (2023).

34 *DRKS - German Clinical Trials Register*, <https://www.drks.de/> (2023).

35 NFDI4Health. *German Central Health Study Hub*, <https://csh.nfdi4health.de/mdr/> (2023).

36 *Metadata portal for observational studies in Nutritional Epidemiology that participated in the INTIMIC project*, <https://mica.mdc-berlin.de/>

37 Bergmann, M. M., Bussas, U. & Boeing, H. Follow-Up Procedures in EPIC-Germany – Data Quality Aspects. *Annals of Nutrition and Metabolism* **43**, 225-234 <https://doi.org/10.1159/000012789> (1999).

38 Kühn, T. *et al.* Albumin, bilirubin, uric acid and cancer risk: results from a prospective population-based study. *British Journal of Cancer* **117**, 1572-1579 <https://doi.org/10.1038/bjc.2017.313> (2017).

39 Li, K., Kaaks, R., Linseisen, J. & Rohrmann, S. Associations of dietary calcium intake and calcium supplementation with myocardial infarction and stroke risk and overall cardiovascular mortality in the Heidelberg cohort of the European Prospective Investigation into Cancer and Nutrition study (EPIC-Heidelberg). *Heart* **98**, 920 <https://doi.org/10.1136/heartjnl-2011-301345> (2012).

40 Nimptsch, K., Rohrmann, S., Kaaks, R. & Linseisen, J. Dietary vitamin K intake in relation to cancer incidence and mortality: results from the Heidelberg cohort of the European Prospective Investigation into Cancer and Nutrition (EPIC-Heidelberg). *The American Journal of Clinical Nutrition* **91**, 1348-1358 <https://doi.org/10.3945/ajcn.2009.28691> (2010).

41 Srour, B. *et al.* Ageing-related markers and risks of cancer and cardiovascular disease: a prospective study in the EPIC-Heidelberg cohort. *European Journal of Epidemiology* **37**, 49-65 <https://doi.org/10.1007/s10654-021-00828-3> (2022).

42 Kharazmi, E., Dossus, L., Rohrmann, S. & Kaaks, R. Pregnancy loss and risk of cardiovascular disease: a prospective population-based cohort study (EPIC-Heidelberg). *Heart* **97**, 49 <https://doi.org/10.1136/hrt.2010.202226> (2011).

43 Li, K., Kaaks, R., Linseisen, J. & Rohrmann, S. Dietary calcium and magnesium intake in relation to cancer incidence and mortality in a German prospective cohort (EPIC-Heidelberg). *Cancer Causes & Control* **22**, 1375 <https://doi.org/10.1007/s10552-011-9810-z> (2011).

44 Li, K., Kaaks, R., Linseisen, J. & Rohrmann, S. Vitamin/mineral supplementation and cancer, cardiovascular, and all-cause mortality in a German prospective cohort (EPIC-Heidelberg). *European Journal of Nutrition* **51**, 407-413 <https://doi.org/10.1007/s00394-011-0224-1> (2012).

45 Boeing, H., Wahrendorf, J. & Becker, N. EPIC-Germany – A Source for Studies into Diet and Risk of Chronic Diseases. *Annals of Nutrition and Metabolism* **43**, 195-204 <https://doi.org/10.1159/000012786> (1999).

46 Braig, S. *et al.* The impact of social status inconsistency on cardiovascular risk factors, myocardial infarction and stroke in the EPIC-Heidelberg cohort. *BMC Public Health* **11**, 104 <https://doi.org/10.1186/1471-2458-11-104> (2011).

47 Lu, D.-L. *et al.* Circulating 27-Hydroxycholesterol and Breast Cancer Risk: Results From the EPIC-Heidelberg Cohort. *JNCI: Journal of the National Cancer Institute* **111**, 365-371 <https://doi.org/10.1093/jnci/djy115> (2019).

48 Li, K. *et al.* Primary preventive potential of major lifestyle risk factors for acute myocardial infarction in men: an analysis of the EPIC-Heidelberg cohort. *European Journal of Epidemiology* **29**, 27-34 <https://doi.org/10.1007/s10654-013-9872-1> (2014).

49 *Deutsches Krebsforschungszentrum in der Helmholtz-Gemeinschaft [German Cancer Research Center in the Helmholtz Association]. EPIC-Heidelberg Study*, <https://www.dkfz.de/de/epidemiologie-krebserkrankungen/arbeitsgr/ernaerepi/EPIC_p03_EPIC_Heidelberg.html#section2>

50 *International Agency for Research on Cancer. European Prospective Investigation into Cancer and Nutrition (EPIC)*, <https://epic.iarc.fr/access/submit_appl_access.php>

51 Weikert, C. *et al.* Joint effects of risk factors for stroke and transient ischemic attack in a German population. *Journal of Neurology* **254**, 315-321 <https://doi.org/10.1007/s00415-006-0358-x> (2007).

52 Schulze, M. B., Hoffmann, K., Kroke, A. & Boeing, H. Risk of Hypertension among Women in the EPIC-Potsdam Study: Comparison of Relative Risk Estimates for Exploratory and Hypothesis-oriented Dietary Patterns. *American Journal of Epidemiology* **158**, 365-373 <https://doi.org/10.1093/aje/kwg156> (2003).

53 Drogan, D., Klipstein-Grobusch, K., Dierkes, J., Weikert, C. & Boeing, H. Dietary intake of folate equivalents and risk of myocardial infarction in the European Prospective Investigation into Cancer and Nutrition (EPIC)–Potsdam study. *Public Health Nutrition* **9**, 465-471 <https://doi.org/10.1079/PHN2005863> (2006).

54 Cabral, M. *et al.* Trace element profile and incidence of type 2 diabetes, cardiovascular disease and colorectal cancer: results from the EPIC-Potsdam cohort study. *European Journal of Nutrition* **60**, 3267-3278 <https://doi.org/10.1007/s00394-021-02494-3> (2021).

55 Galbete, C. *et al.* Nordic diet, Mediterranean diet, and the risk of chronic diseases: the EPIC-Potsdam study. *BMC Medicine* **16**, 99 <https://doi.org/10.1186/s12916-018-1082-y> (2018).

56 von Ruesten, A., Weikert, C., Fietze, I. & Boeing, H. Association of Sleep Duration with Chronic Diseases in the European Prospective Investigation into Cancer and Nutrition (EPIC)-Potsdam Study. *PLOS ONE* **7**, e30972 <https://doi.org/10.1371/journal.pone.0030972> (2012).

57 Spranger, J. *et al.* Inflammatory Cytokines and the Risk to Develop Type 2 Diabetes: Results of the Prospective Population-Based European Prospective Investigation into Cancer and Nutrition (EPIC)-Potsdam Study. *Diabetes* **52**, 812-817 <https://doi.org/10.2337/diabetes.52.3.812> (2003).

58 Kroke, A. *et al.* Blood pressure measurement in epidemiological studies: a comparative analysis of two methods. Data from the EPIC-Potsdam Study. *Journal of Hypertension* **16** (1998).

59 Heidemann, C. *et al.* Association of a diabetes risk score with risk of myocardial infarction, stroke, specific types of cancer, and mortality: a prospective study in the European Prospective Investigation into Cancer and Nutrition (EPIC)-Potsdam cohort. *European Journal of Epidemiology* **24**, 281-288 <https://doi.org/10.1007/s10654-009-9338-7> (2009).

60 *German Institute of Human Nutrition Potsdam-Rehbrücke (DIfE). EPIC-Potsdam Study*, <https://www.dife.de/en/research/cooperations/epic-study/>

61 Lange, C. *et al.* Data Resource Profile: German Health Update (GEDA)—the health interview survey for adults in Germany. *International Journal of Epidemiology* **44**, 442-450 <https://doi.org/10.1093/ije/dyv067> (2015).

62 Fuchs, J., Busch, M., Lange, C. & Scheidt-Nave, C. Prevalence and patterns of morbidity among adults in Germany. *Bundesgesundheitsblatt - Gesundheitsforschung - Gesundheitsschutz* **55**, 576-586 <https://doi.org/10.1007/s00103-012-1464-9> (2012).

63 Gesundheitliche Lage der Bevölkerung in Deutschland [Health situation of the population in Germany]. *Journal of Health Monitoring* **1/2017**

64 Allen, J. *et al.* Gesundheit in Deutschland aktuell (GEDA 2019/2020-EHIS)-Hintergrund und Methodik [German Health Update (GEDA 2019/2020-EHIS)-Background and Methods]. (2021).

65 Robert Koch Institute. *GEDA: Gesundheit Deutschland aktuell [GEDA: German Health Update]*, <https://www.rki.de/DE/Content/Gesundheitsmonitoring/Studien/Geda/Geda_node.html;jsessionid=2BBA9A847CFD1BBA1700B1850FC469D5.internet101>

66 *GEDA: Gesundheit in Deutschland aktuell [GEDA: German Health Update]*, <https://www.geda-studie.de/de/deutsch/ergebnisse/geda-20142015-ehis.html>

67 Wild, P. S. *et al.* Die Gutenberg Gesundheitsstudie [The Gutenberg Health Study]. *Bundesgesundheitsblatt - Gesundheitsforschung - Gesundheitsschutz* **55**, 824-830 <https://doi.org/10.1007/s00103-012-1502-7> (2012).

68 Hegewald, J. *et al.* Work-life conflict and cardiovascular health: 5-year follow-up of the Gutenberg Health Study. *PLOS ONE* **16**, e0251260 <https://doi.org/10.1371/journal.pone.0251260> (2021).

69 Rossnagel, K. *et al.* Long working hours and risk of cardiovascular outcomes and diabetes type II: five-year follow-up of the Gutenberg Health Study (GHS). *International Archives of Occupational and Environmental Health* **95**, 303-312 <https://doi.org/10.1007/s00420-021-01786-9> (2022).

70 Schnabel, R. B. *et al.* Non-invasive peripheral vascular function, incident cardiovascular disease, and mortality in the general population. *Cardiovascular Research* **118**, 904-912 <https://doi.org/10.1093/cvr/cvab087> (2022).

71 Panova-Noeva, M. *et al.* Coagulation and inflammation in long-term cancer survivors: results from the adult population. *Journal of Thrombosis and Haemostasis* **16**, 699-708 <https://doi.org/10.1111/jth.13975> (2018).

72 Reiner, I. C. *et al.* The association of chronic anxiousness with cardiovascular disease and mortality in the community: results from the Gutenberg Health Study. *Scientific Reports* **10**, 12436 <https://doi.org/10.1038/s41598-020-69427-8> (2020).

73 Wild, P. S. *et al.* Distribution and Categorization of Left Ventricular Measurements in the General Population. *Circulation: Cardiovascular Imaging* **3**, 604-613 <https://doi.org/10.1161/CIRCIMAGING.109.911933> (2010).

74 Grossmann, V. *et al.* Profile of the Immune and Inflammatory Response in Individuals With Prediabetes and Type 2 Diabetes. *Diabetes Care* **38**, 1356-1364 <https://doi.org/10.2337/dc14-3008> (2015).

75 Prochaska, J. H. *et al.* Chronic venous insufficiency, cardiovascular disease, and mortality: a population study. *European Heart Journal* **42**, 4157-4165 <https://doi.org/10.1093/eurheartj/ehab495> (2021).

76 Schnabel, R. B. *et al.* Multiple Biomarkers and Atrial Fibrillation in the General Population. *PLOS ONE* **9**, e112486 <https://doi.org/10.1371/journal.pone.0112486> (2014).

77 Raum, P. *et al.* Prevalence and Cardiovascular Associations of Diabetic Retinopathy and Maculopathy: Results from the Gutenberg Health Study. *PLOS ONE* **10**, e0127188 <https://doi.org/10.1371/journal.pone.0127188> (2015).

78 Münzel, T. *et al.* Heart rate, mortality, and the relation with clinical and subclinical cardiovascular diseases: results from the Gutenberg Health Study. *Clinical Research in Cardiology* **108**, 1313-1323 <https://doi.org/10.1007/s00392-019-01466-2> (2019).

79 Schmitt, V. H. *et al.* Cardiovascular profiling in the diabetic continuum: results from the population-based Gutenberg Health Study. *Clinical Research in Cardiology* **111**, 272-283 <https://doi.org/10.1007/s00392-021-01879-y> (2022).

80 Schnabel, R. B., Johannsen, S. S., Wild, P. S. & Blankenberg, S. Prävalenz und Risikofaktoren von Vorhofflimmern in Deutschland [Prevalence and risk factors of atrial fibrillation in Germany]. *Herz* **40**, 8-15 <https://doi.org/10.1007/s00059-014-4199-6> (2015).

81 Baum, C. *et al.* Subclinical impairment of lung function is related to mild cardiac dysfunction and manifest heart failure in the general population. *International Journal of Cardiology* **218**, 298-304 <https://doi.org/10.1016/j.ijcard.2016.05.034> (2016).

82 Börschel, C. S. *et al.* Noninvasive peripheral vascular function and atrial fibrillation in the general population. *Journal of Hypertension* **37** <https://doi.org/10.1097/HJH.0000000000002000> (2019).

83 *Universitätsmedizin Mainz. Gutenberg Health Study*, <http://www.gutenberghealthstudy.org/ghs/overview.html?L=1>

84 *Universitätsmedizin Mainz. Gutenberg-Gesundheitsstudie (GHS) [Gutenberg Health Study (GHS)]*, <https://www.unimedizin-mainz.de/pkmp-cesm/forschung-studien/studien-biodatenbanken/gutenberg-gesundheitsstudie.html>

85 *synchros.eu cohort repository*, <https://synchros.eu/>

86 *Das Gesundheitswesen. Sonderheft 2 (Schwerpunktheft zum Bundes-Gesundheitssurvey 1998) [Healthcare. Special issue 2 (special issue for the German National Health Interview and Examination Survey 1998)] Volume 61, December 1999*, <https://www.thieme.de/statics/dokumente/thieme/final/de/dokumente/zw_das-gesundheitswesen/gesu-suppl_klein.pdf>

87 Robert Koch Institute. *BGS98: Bundes-Gesundheitssurvey 1998 [GNHIES98: the German National Health Interview and Examination Survey 1998]*, <https://www.rki.de/DE/Content/Gesundheitsmonitoring/Studien/Degs/bgs98/bgs98_node.html;jsessionid=2C012A0D7691B05444747F0ADD205223.internet082>

88 Jagodzinski, A. *et al.* Rationale and Design of the Hamburg City Health Study. *European Journal of Epidemiology* **35**, 169-181 <https://doi.org/10.1007/s10654-019-00577-4> (2020).

89 Kotin, J. *et al.* Association between periodontitis and metabolic syndrome in the Hamburg City Health Study. *Journal of Periodontology* **93**, 1150-1160 <https://doi.org/10.1002/JPER.21-0464> (2022).

90 Lamprecht, R. *et al.* Cross-sectional analysis of the association of periodontitis with carotid intima media thickness and atherosclerotic plaque in the Hamburg City health study. *Journal of Periodontal Research* **57**, 824-834 <https://doi.org/10.1111/jre.13021> (2022).

91 Struppek, J. *et al.* Periodontitis, dental plaque, and atrial fibrillation in the Hamburg City Health Study. *PLOS ONE* **16**, e0259652 <https://doi.org/10.1371/journal.pone.0259652> (2021).

92 *Hamburg City Health Study*, <http://hchs.hamburg/>

93 *euCanSHare: An EU-Canada joint infrastructure for next-generation multi-Study Heart research*, <https://eucanshare.bsc.es/platform/> (2020).

94 *Maelstrom Research* <https://www.maelstrom-research.org/> (2021).

95 U.S. National Library of Medicine. *ClinicalTrials.gov*, <https://clinicaltrials.gov/> (2023).

96 Bokhof, B., Eisele, L., Erbel, R. & Moebus, S. Agreement between different survey instruments to assess incident and prevalent tumors and medical records – results of the Heinz Nixdorf Recall Study. *Cancer Epidemiology* **38**, 181-192 <https://doi.org/10.1016/j.canep.2014.01.002> (2014).

97 Erbel, R. *et al.* Die Heinz Nixdorf Recall Studie [The Heinz Nixdorf Recall Study]. *Bundesgesundheitsblatt-Gesundheitsforschung-Gesundheitsschutz* **55**, 809-815 <https://doi.org/10.1007/s00103-012-1490-7> (2012).

98 Kröger, K. *et al.* Prevalence of Peripheral Arterial Disease – Results of the Heinz Nixdorf Recall Study. *European Journal of Epidemiology* **21**, 279 <https://doi.org/10.1007/s10654-006-0015-9> (2006).

99 Mahabadi, A. A. *et al.* Association of bilirubin with coronary artery calcification and cardiovascular events in the general population without known liver disease: the Heinz Nixdorf Recall study. *Clinical Research in Cardiology* **103**, 647-653 <https://doi.org/10.1007/s00392-014-0697-z> (2014).

100 Kara, K. *et al.* NT-proBNP is superior to BNP for predicting first cardiovascular events in the general population: The Heinz Nixdorf Recall Study. *International Journal of Cardiology* **183**, 155-161 <https://doi.org/10.1016/j.ijcard.2015.01.082> (2015).

101 Hoffmann, B. *et al.* Air Quality, Stroke, and Coronary Events. *Dtsch Arztebl International* **112**, 195-201 <https://doi.org/10.3238/arztebl.2015.0195> (2015).

102 Schmermund, A. *et al.* Assessment of clinically silent atherosclerotic disease and established and novel risk factors for predicting myocardial infarction and cardiac death in healthy middle-aged subjects: Rationale and design of the Heinz Nixdorf RECALL Study. *American Heart Journal* **144**, 212-218 <https://doi.org/10.1067/mhj.2002.123579> (2002).

103 Horacek, M. *et al.* Prävalenz der arteriellen Hypertonie in der westdeutschen Bevölkerung [Prevalence of arterial hypertension in the West German population]. *Herz* **37**, 721-727 <https://doi.org/10.1007/s00059-012-3684-z> (2012).

104 Icks, A. *et al.* High Depressive Symptoms in Previously Undetected Diabetes - 10-Year Follow-Up Results of the Heinz Nixdorf Recall Study. *Clin Epidemiol* **13**, 429-438 <https://doi.org/10.2147/clep.S294342> (2021).

105 Icks, A. *et al.* Diabetes incidence does not differ between subjects with and without high depressive symptoms — 5-year follow-up results of the Heinz Nixdorf Recall Study. *Diabetic Medicine* **30**, 65-69 <https://doi.org/10.1111/j.1464-5491.2012.03724.x> (2013).

106 Behrens, T. *et al.* Shift work and the incidence of prostate cancer: a 10-year follow-up of a German population-based cohort study. *Scandinavian Journal of Work, Environment & Health* **43**, 560-568 <https://doi.org/10.5271/sjweh.3516> (2017).

107 Lehmann, N. *et al.* Value of Progression of Coronary Artery Calcification for Risk Prediction of Coronary and Cardiovascular Events. *Circulation* **137**, 665-679 <https://doi.org/10.1161/CIRCULATIONAHA.116.027034> (2018).

108 *Heinz Nixdorf Recall Studie [Heinz Nixdorf Recall Study]*, <https://www.uni-due.de/recall-studie/die-studien/hnr/>

109 *German Biobank Registry. TMF e.V*, <https://www.tmf-ev.de/BiobankenRegisterEN_Alt/Registry.aspx>

110 Ahrens, W. *et al.* Cohort Profile: The transition from childhood to adolescence in European children–how I.Family extends the IDEFICS cohort. *International Journal of Epidemiology* **46**, 1394-1395j <https://doi.org/10.1093/ije/dyw317> (2017).

111 Bammann, K., Lissner, L., Pigeot, I. & Ahrens, W. *Instruments for health surveys in children and adolescents*. (Springer International Publishing, 2019. (See also <https://www.bips-institut.de/en/pages/ifhs.html>; accessed 03 Aug 2022)).

112 Ahrens, W. *et al.* The IDEFICS cohort: design, characteristics and participation in the baseline survey. *International Journal of Obesity* **35**, S3-S15 <https://doi.org/10.1038/ijo.2011.30> (2011).

113 de Moraes, A. C. F. *et al.* Incidence of high blood pressure in children — Effects of physical activity and sedentary behaviors: The IDEFICS study: High blood pressure, lifestyle and children. *International Journal of Cardiology* **180**, 165-170 <https://doi.org/10.1016/j.ijcard.2014.11.175> (2015).

114 Ahrens, W. *et al.* Metabolic syndrome in young children: definitions and results of the IDEFICS study. *International Journal of Obesity* **38**, S4-S14 <https://doi.org/10.1038/ijo.2014.130> (2014).

115 *IDEFICS - Identification and prevention of Dietary - and lifestyle-induced health EFfects In Children and infantS*, <https://www.ideficsstudy.eu/index.php?id=1148&L=530%27>

116 *I.Family. IDEFICS/I.Family follow-up study - lifestyle and health*, <https://www.ifamilystudy.eu/>

117 *NFDI4Health Task Force COVID-19 Study Portal*, <https://covid19.studyhub.nfdi4health.de/>

118 BMC. *ISRCTN registry*, <https://www.isrctn.com/> (2023).

119 Peters, A. *et al.* Multimorbidität und erfolgreiches Altern [Multimorbidity and successful aging]. *Zeitschrift für Gerontologie und Geriatrie [Journal for gerontology and geriatrics]* **44**, 41-54 <https://doi.org/10.1007/s00391-011-0245-7> (2011).

120 Mühlenbruch, K. *et al.* Update of the German Diabetes Risk Score and external validation in the German MONICA/KORA study. *Diabetes Research and Clinical Practice* **104**, 459-466 <https://doi.org/10.1016/j.diabres.2014.03.013> (2014).

121 Rathmann, W. *et al.* High prevalence of undiagnosed diabetes mellitus in Southern Germany: Target populations for efficient screening. The KORA survey 2000. *Diabetologia* **46**, 182-189 <https://doi.org/10.1007/s00125-002-1025-0> (2003).

122 Meisinger, C., Koenig, W., Baumert, J. & Döring, A. Uric Acid Levels Are Associated With All-Cause and Cardiovascular Disease Mortality Independent of Systemic Inflammation in Men From the General Population. *Arteriosclerosis, Thrombosis, and Vascular Biology* **28**, 1186-1192 <https://doi.org/10.1161/ATVBAHA.107.160184> (2008).

123 Seyed Khoei, N., Anton, G., Peters, A., Freisling, H. & Wagner, K.-H. The Association between Serum Bilirubin Levels and Colorectal Cancer Risk: Results from the Prospective Cooperative Health Research in the Region of Augsburg (KORA) Study in Germany. *Antioxidants* **9** <https://doi.org/10.3390/antiox9100908> (2020).

124 Löwel, H., Meisinger, C., Heier, M. & Hörmann, A. The Population-Based Acute Myocardial Infarction (AMI) Registry of the MONICA/KORA Study Region of Augsburg. *Gesundheitswesen* **67**, 31-37 <https://doi.org/10.1055/s-2005-858241> (2005).

125 Holle, R., Happich, M., Löwel, H., Wichmann, H. E. & for the, M. K. S. G. KORA - A Research Platform for Population Based Health Research. *Gesundheitswesen* **67**, 19-25 <https://doi.org/10.1055/s-2005-858235> (2005).

126 Lorbeer, R. *et al.* Association of antecedent cardiovascular risk factor levels and trajectories with cardiovascular magnetic resonance-derived cardiac function and structure. *Journal of Cardiovascular Magnetic Resonance* **23**, 2 <https://doi.org/10.1186/s12968-020-00698-w> (2021).

127 Arshadipour, A. *et al.* Impact of prenatal and childhood adversity effects around World War II on multimorbidity: results from the KORA-Age study. *BMC Geriatrics* **22**, 115 <https://doi.org/10.1186/s12877-022-02793-2> (2022).

128 Bamberg, F. *et al.* Subclinical Disease Burden as Assessed by Whole-Body MRI in Subjects With Prediabetes, Subjects With Diabetes, and Normal Control Subjects From the General Population: The KORA-MRI Study. *Diabetes* **66**, 158-169 <https://doi.org/10.2337/db16-0630> (2017).

129 Helmholtz Zentrum München. *KORA - Kooperative Gesundheitsforschung in der Region Ausburg [KORA - The Cooperative Health Research in the Region of Augsburg]*, <https://www.helmholtz-muenchen.de/kora/index.html>

130 The MORGAM Project. *Description of MORGAM Cohorts*, <https://www.thl.fi/morgam/index.html>

131 *The German Biobank Directory*, <https://www.bbmri.de/>

132 *KORA.PASST: Project Application Self-Service Tool*, <https://helmholtz-muenchen.managed-otrs.com/external>

133 Hasselhorn, H. M. *et al.* Cohort profile: The lidA Cohort Study—a German Cohort Study on Work, Age, Health and Work Participation. *International Journal of Epidemiology* **43**, 1736-1749 <https://doi.org/10.1093/ije/dyu021> (2014).

134 Bergische Universität Wuppertal. *lidA - leben in der Arbeit [lidA - German Cohort Study on Work, Age, Health and Work Participation]*, <https://arbeit.uni-wuppertal.de/de/studie/>

135 *Forschungsdatenzentrum der Bundesagentur für Arbeit im In stitut für Arbeitsmarkt- und Berufsforschung [Research data center of the Federal Employment Agency in the Institute for Labor Market and Vocational Research]. lidA - Survey Data*, <https://fdz.iab.de/en/our-data-products/archived-data/lida/>

136 Loeffler, M. *et al.* The LIFE-Adult-Study: objectives and design of a population-based cohort study with 10,000 deeply phenotyped adults in Germany. *BMC Public Health* **15**, 691 <https://doi.org/10.1186/s12889-015-1983-z> (2015).

137 Engel, C. *et al.* Cohort Profile: The LIFE-Adult-Study. *International Journal of Epidemiology*, dyac114 <https://doi.org/10.1093/ije/dyac114> (2022).

138 Buchmann, N. *et al.* Association between lipoprotein(a) level and type 2 diabetes: no evidence for a causal role of lipoprotein(a) and insulin. *Acta Diabetologica* **54**, 1031-1038 <https://doi.org/10.1007/s00592-017-1036-4> (2017).

139 Diseases, L. L. R. C. f. C. *LIFE-Adult*, <https://life.uni-leipzig.de/en/adults/life_adult.html>

140 Leipzig Health Atlas. *LIFE Adult*, <https://www.health-atlas.de/projects/5>

141 *LIFE-Datenportal [LIFE data portal]*, <https://ldp.life.uni-leipzig.de/>

142 German National Cohort Consortium. The German National Cohort: aims, study design and organization. *European Journal of Epidemiology* **29**, 371-382 <https://doi.org/10.1007/s10654-014-9890-7> (2014).

143 Ahrens, W., Greiser, K. H., Linseisen, J., Pischon, T. & Pigeot, I. Erforschung von Erkrankungen in der NAKO Gesundheitsstudie. Die wichtigsten gesundheitlichen Endpunkte und ihre Erfassung [Research into diseases in the NAKO health study. The most important health endpoints and their recording]. *Bundesgesundheitsblatt - Gesundheitsforschung - Gesundheitsschutz* **63**, 376-384 <https://doi.org/10.1007/s00103-020-03111-0> (2020).

144 Jaeschke, L. *et al.* Erfassung selbst berichteter kardiovaskulärer und metabolischer Erkrankungen in der NAKO Gesundheitsstudie: Methoden und erste Ergebnisse [Collecting self-reported cardiovascular and metabolic diseases in the NAKO health study: methods and first results]. *Bundesgesundheitsblatt - Gesundheitsforschung - Gesundheitsschutz* **63**, 439-451 <https://doi.org/10.1007/s00103-020-03108-9> (2020).

145 Nimptsch, K. *et al.* Selbstberichtete Krebserkrankungen in der NAKO Gesundheitsstudie: Erfassungsmethoden und erste Ergebnisse [Self-reported cancers in the NAKO health study: collection methods and first results]. *Bundesgesundheitsblatt - Gesundheitsforschung - Gesundheitsschutz* **63**, 385-396 <https://doi.org/10.1007/s00103-020-03113-y> (2020).

146 *NAKO Gesundheitsstudie [NAKO: German National Cohort]*, <https://nako.de/>

147 *re3data.org: Registry of Research Data Repositories*, <https://www.re3data.org/> (2023).

148 *NAKO Transferhub*, <https://transfer.nako.de/transfer/index>

149 Völzke, H. Study of Health in Pomerania (SHIP). *Bundesgesundheitsblatt - Gesundheitsforschung - Gesundheitsschutz* **55**, 790-794 <https://doi.org/10.1007/s00103-012-1483-6> (2012).

150 Völzke, H. *et al.* Cohort Profile Update: The Study of Health in Pomerania (SHIP). *International Journal of Epidemiology*, dyac034 <https://doi.org/10.1093/ije/dyac034> (2022).

151 Angelow, A., Reber, K. C., Schmidt, C. O., Baumeister, S. E. & Chenot, J.-F. Untersuchung der Prävalenz kardiologischer Risikofaktoren in der Allgemeinbevölkerung: Ein Vergleich ambulanter ärztlicher Abrechnungsdaten mit Daten einer populationsbasierten Studie [Investigating the prevalence of cardiological risk factors in the general population: A comparison of outpatient medical billing data with data from a population-based study]. *Gesundheitswesen* **81**, 791-800 <https://doi.org/10.1055/a-0588-4736> (2019).

152 Schmidt, C. O. *et al.* Die Integration von Primär- und Sekundärdaten in der Study of Health in Pomerania und die Beschreibung von klinischen Endpunkten am Beispiel Schlaganfall [The integration of primary and secondary data in the Study of Health in Pomerania and the description of clinical endpoints using stroke as an example]. *Gesundheitswesen* **77**, e20-e25 <https://doi.org/10.1055/s-0034-1395648> (2015).

153 Schwedhelm, E. *et al.* Incidence of All-Cause and Cardiovascular Mortality Predicted by Symmetric Dimethylarginine in the Population-Based Study of Health in Pomerania. *PLOS ONE* **9**, e96875 <https://doi.org/10.1371/journal.pone.0096875> (2014).

154 Völzke, H. *et al.* Cohort Profile: The Study of Health in Pomerania. *International Journal of Epidemiology* **40**, 294-307 <https://doi.org/10.1093/ije/dyp394> (2011).

155 Schipf, S. *et al.* Low total testosterone is associated with increased risk of incident type 2 diabetes mellitus in men: results from the Study of Health in Pomerania (SHIP). *The Aging Male* **14**, 168-175 <https://doi.org/10.3109/13685538.2010.524955> (2011).

156 Völzke, H. *et al.* A new, accurate predictive model for incident hypertension. *Journal of Hypertension* **31** <https://doi.org/10.1097/HJH.0b013e328364a16d> (2013).

157 Hoffmann, W. *et al.* Not Just the Demographic Change – The Impact of Trends in Risk Factor Prevalences on the Prediction of Future Cases of Myocardial Infarction. *PLOS ONE* **10**, e0131256 <https://doi.org/10.1371/journal.pone.0131256> (2015).

158 Friedrich, N. *et al.* Correlates of Adverse Outcomes in Abdominally Obese Individuals: Findings from the Five-Year Followup of the Population-Based Study of Health in Pomerania. *Journal of Obesity* **2013**, 762012 <https://doi.org/10.1155/2013/762012> (2013).

159 Ittermann, T. *et al.* Hyperthyroxinemia is positively associated with prevalent and incident type 2 diabetes mellitus in two population-based samples from Northeast Germany and Denmark. *Nutrition, Metabolism and Cardiovascular Diseases* **28**, 173-179 <https://doi.org/10.1016/j.numecd.2017.10.016> (2018).

160 Moeller, M. *et al.* Mortality is associated with inflammation, anemia, specific diseases and treatments, and molecular markers. *PLOS ONE* **12**, e0175909 <https://doi.org/10.1371/journal.pone.0175909> (2017).

161 Völzke, H. *et al.* Prevalence Trends in Lifestyle-Related Risk Factors. *Dtsch Arztebl International* **112**, 185-192 <https://doi.org/10.3238/arztebl.2015.0185> (2015).

162 Markus, M. R. P. *et al.* Prediabetes is associated with lower brain gray matter volume in the general population. The Study of Health in Pomerania (SHIP). *Nutrition, Metabolism and Cardiovascular Diseases* **27**, 1114-1122 <https://doi.org/10.1016/j.numecd.2017.10.007> (2017).

163 Rotheudt, L. *et al.* Sphingosine-1-phosphate and vascular disease in the general population. *Atherosclerosis* **350**, 73-81 <https://doi.org/10.1016/j.atherosclerosis.2022.03.020> (2022).

164 Markus, M. R. P. *et al.* Association between hepatic steatosis and serum liver enzyme levels with atrial fibrillation in the general population: The Study of Health in Pomerania (SHIP). *Atherosclerosis* **245**, 123-131 <https://doi.org/10.1016/j.atherosclerosis.2015.12.023> (2016).

165 Markus, M. R. P. *et al.* Light to Moderate Alcohol Consumption Is Associated With Lower Risk of Aortic Valve Sclerosis. *Arteriosclerosis, Thrombosis, and Vascular Biology* **35**, 1265-1270 <https://doi.org/10.1161/ATVBAHA.114.304831> (2015).

166 Richter, A. *et al.* The effects of incidental findings from whole-body MRI on the frequency of biopsies and detected malignancies or benign conditions in a general population cohort study. *European Journal of Epidemiology* **35**, 925-935 <https://doi.org/10.1007/s10654-020-00679-4> (2020).

167 University of Greifswald. *SHIP - Study of Health in Pomerania*, <https://www2.medizin.uni-greifswald.de/cm/fv/ship/>

168 *Ernst-Moritz-Arndt-Universität Greifswald, Medizinische Fakultät. FVCM Transferstelle für Daten und Biomaterialien [Transfer unit for data and biomaterials]*, <https://www.fvcm.med.uni-greifswald.de/>

169 Wilkinson, M. D. *et al.* The FAIR Guiding Principles for scientific data management and stewardship. *Scientific Data* **3**, 160018 <https://doi.org/10.1038/sdata.2016.18> (2016).

#
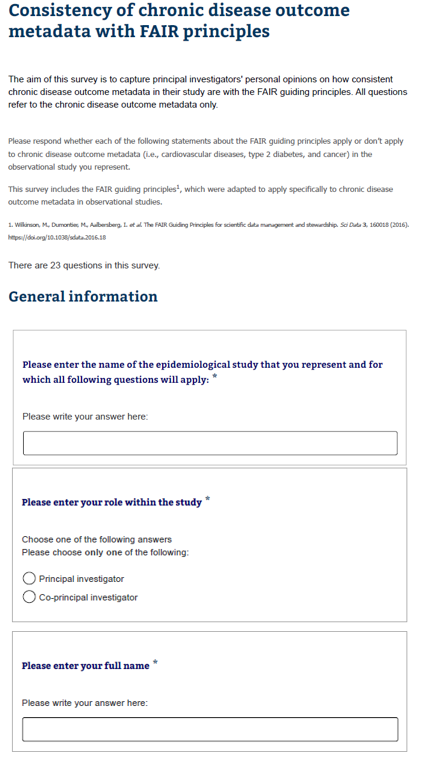
Supplementary Figure 1: Principal investigator survey


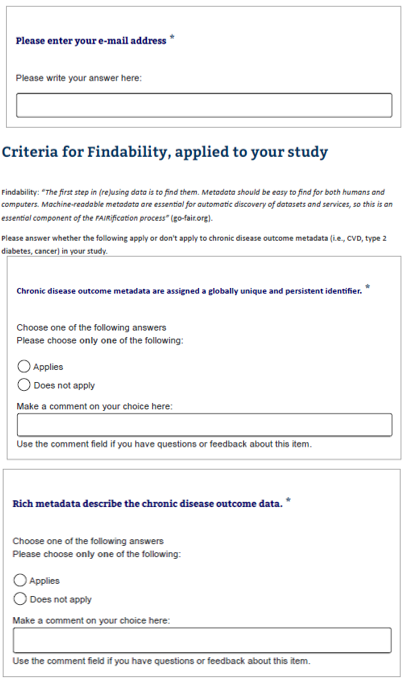


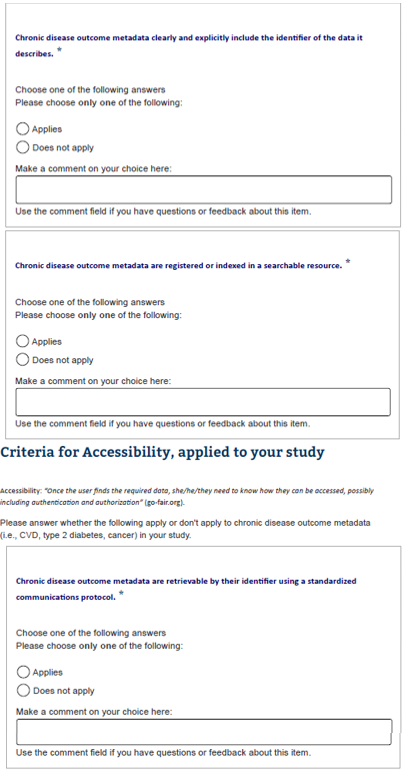

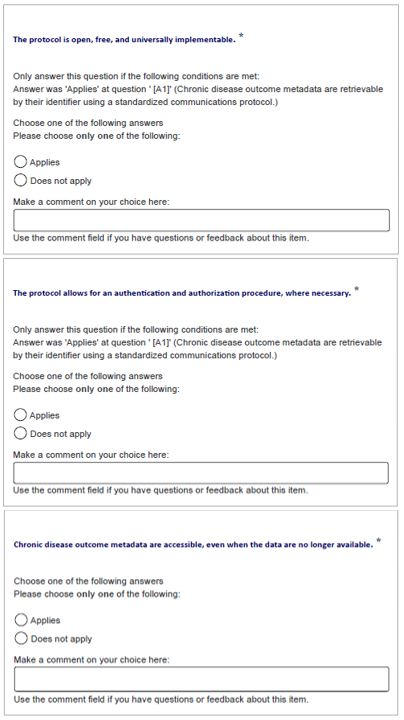


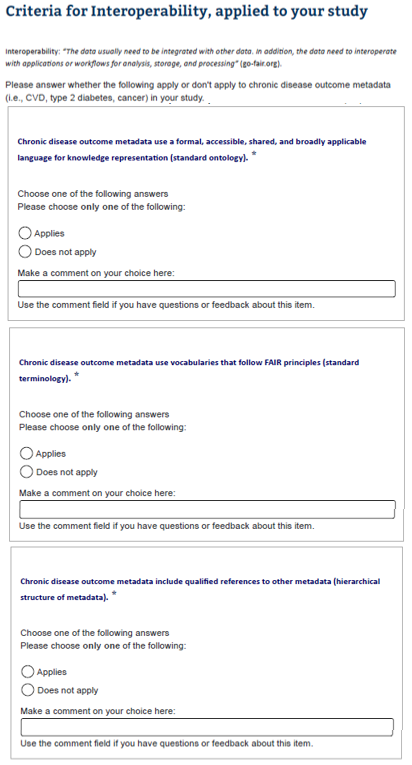

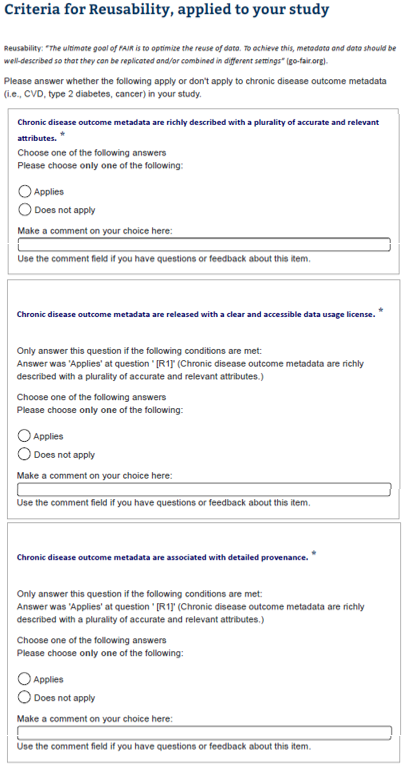


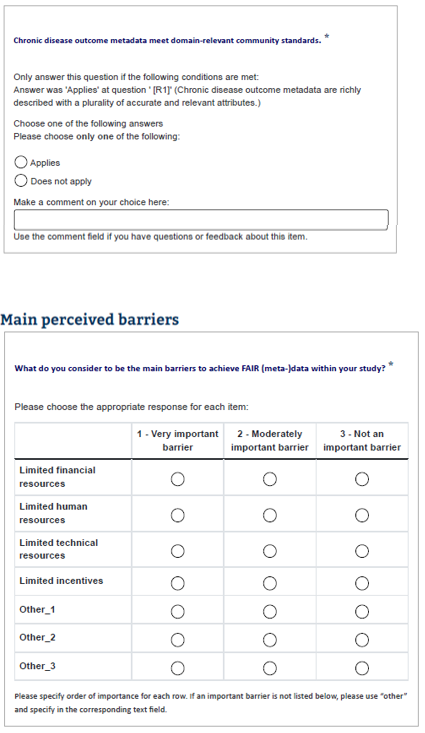

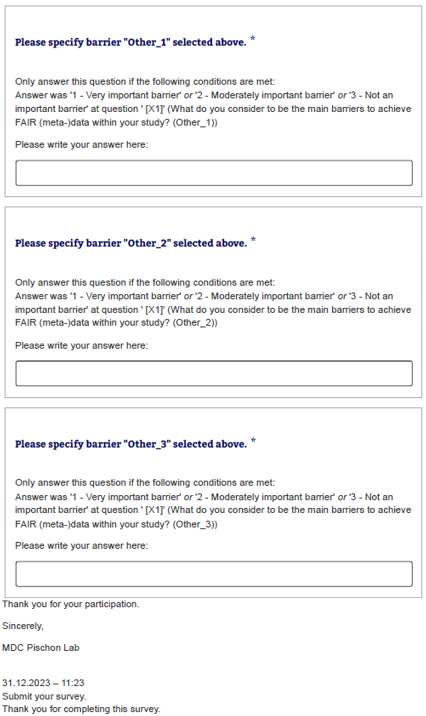

Supplement: Supplementary file 1 — Supplementary Information [file 41597_2023_2726_MOESM1_ESM.docx]
